# Pathology of Tumors Associated With Pathogenic Germline Variants in 9 Breast Cancer Susceptibility Genes

**DOI:** 10.1001/jamaoncol.2021.6744

**Published:** 2022-01-27

**Authors:** Breast Cancer Association Consortium, Nasim Mavaddat, Leila Dorling, Sara Carvalho, Jamie Allen, Anna González-Neira, Renske Keeman, Manjeet K. Bolla, Joe Dennis, Qin Wang, Thomas U. Ahearn, Irene L. Andrulis, Matthias W. Beckmann, Sabine Behrens, Javier Benitez, Marina Bermisheva, Carl Blomqvist, Natalia V. Bogdanova, Stig E. Bojesen, Ignacio Briceno, Thomas Brüning, Nicola J. Camp, Archie Campbell, Jose E. Castelao, Jenny Chang-Claude, Stephen J. Chanock, Georgia Chenevix-Trench, Hans Christiansen, Kamila Czene, Thilo Dörk, Mikael Eriksson, D. Gareth Evans, Peter A. Fasching, Jonine D. Figueroa, Henrik Flyger, Marike Gabrielson, Manuela Gago-Dominguez, Jürgen Geisler, Graham G. Giles, Pascal Guénel, Andreas Hadjisavvas, Eric Hahnen, Per Hall, Ute Hamann, Jaana M. Hartikainen, Mikael Hartman, Reiner Hoppe, Anthony Howell, Anna Jakubowska, Audrey Jung, Elza K. Khusnutdinova, Vessela N. Kristensen, Jingmei Li, Swee Ho Lim, Annika Lindblom, Maria A. Loizidou, Artitaya Lophatananon, Jan Lubiński, Michael J. Madsen, Arto Mannermaa, Mehdi Manoochehri, Sara Margolin, Dimitrios Mavroudis, Roger L. Milne, Nur Aishah Mohd Taib, Anna Morra, Kenneth Muir, Nadia Obi, Ana Osorio, Tjoung-Won Park-Simon, Paolo Peterlongo, Paolo Radice, Emmanouil Saloustros, Elinor J. Sawyer, Rita K. Schmutzler, Mitul Shah, Xueling Sim, Melissa C. Southey, Heather Thorne, Ian Tomlinson, Diana Torres, Thérèse Truong, Cheng Har Yip, Amanda B. Spurdle, Maaike P.G. Vreeswijk, Alison M. Dunning, Montserrat García-Closas, Paul D.P. Pharoah, Anders Kvist, Taru A. Muranen, Heli Nevanlinna, Soo Hwang Teo, Peter Devilee, Marjanka K. Schmidt, Douglas F. Easton

**Affiliations:** 1 Centre for Cancer Genetic Epidemiology, Department of Public Health and Primary Care, University of Cambridge, Cambridge, England; 2 Human Cancer Genetics Programme, Spanish National Cancer Research Centre, Madrid, Spain; 3 Division of Molecular Pathology, the Netherlands Cancer Institute–Antoni van Leeuwenhoek Hospital, Amsterdam, the Netherlands; 4 Division of Cancer Epidemiology and Genetics, National Cancer Institute, National Institutes of Health, Department of Health and Human Services, Bethesda, Maryland; 5 Fred A. Litwin Center for Cancer Genetics, Lunenfeld-Tanenbaum Research Institute of Mount Sinai Hospital, Toronto, Ontario, Canada; 6 Department of Molecular Genetics, University of Toronto, Toronto, Ontario, Canada; 7 Department of Gynecology and Obstetrics, Comprehensive Cancer Center Erlangen-EMN, University Hospital Erlangen, Friedrich-Alexander University Erlangen-Nuremberg, Erlangen, Germany; 8 Division of Cancer Epidemiology, German Cancer Research Center, Heidelberg, Germany; 9 Biomedical Network on Rare Diseases, Madrid, Spain; 10 Institute of Biochemistry and Genetics, Ufa Federal Research Centre of the Russian Academy of Sciences, Ufa, Russia; 11 Department of Oncology, Helsinki University Hospital, University of Helsinki, Helsinki, Finland; 12 Department of Radiation Oncology, Hannover Medical School, Hannover, Germany; 13 Gynaecology Research Unit, Hannover Medical School, Hannover, Germany; 14 N.N. Alexandrov Research Institute of Oncology and Medical Radiology, Minsk, Belarus; 15 Copenhagen General Population Study, Herlev and Gentofte Hospital, Copenhagen University Hospital, Herlev, Denmark; 16 Department of Clinical Biochemistry, Herlev and Gentofte Hospital, Copenhagen University Hospital, Herlev, Denmark; 17 Faculty of Health and Medical Sciences, University of Copenhagen, Copenhagen, Denmark; 18 Medical Faculty, Universidad de La Sabana, Bogota, Colombia; 19 Institute for Prevention and Occupational Medicine of the German Social Accident Insurance, Institute of the Ruhr University Bochum, Bochum, Germany; 20 Department of Internal Medicine and Huntsman Cancer Institute, University of Utah, Salt Lake City; 21 Centre for Genomic and Experimental Medicine, Institute of Genetics & Cancer, University of Edinburgh, Edinburgh, Scotland; 22 Usher Institute of Population Health Sciences and Informatics, University of Edinburgh, Edinburgh, Scotland; 23 Oncology and Genetics Unit, Instituto de Investigación Sanitaria Galicia Sur, Xerencia de Xestion Integrada de Vigo-SERGAS, Vigo, Spain; 24 Cancer Epidemiology Group, University Cancer Center Hamburg, University Medical Center Hamburg-Eppendorf, Hamburg, Germany; 25 Department of Genetics and Computational Biology, QIMR Berghofer Medical Research Institute, Brisbane, Queensland, Australia; 26 Department of Medical Epidemiology and Biostatistics, Karolinska Institutet, Stockholm, Sweden; 27 Division of Evolution and Genomic Sciences, School of Biological Sciences, Faculty of Biology, Medicine and Health, University of Manchester, Manchester Academic Health Science Centre, Manchester, England; 28 North West Genomics Laboratory Hub, Manchester Centre for Genomic Medicine, St Mary’s Hospital, Manchester University National Health Service Foundation Trust, Manchester Academic Health Science Centre, Manchester, England; 29 David Geffen School of Medicine, Department of Medicine Division of Hematology and Oncology, University of California at Los Angeles; 30 Cancer Research UK Edinburgh Centre, University of Edinburgh, Edinburgh, Scotland; 31 Department of Breast Surgery, Herlev and Gentofte Hospital, Copenhagen University Hospital, Herlev, Denmark; 32 Fundación Pública Galega de Medicina Xenómica, Instituto de Investigación Sanitaria de Santiago de Compostela, Complejo Hospitalario Universitario de Santiago, SERGAS, Santiago de Compostela, Spain; 33 Moores Cancer Center, University of California San Diego, La Jolla; 34 Department of Oncology, Akershus University Hospital, Lørenskog, Norway; 35 Institute of Clinical Medicine, Faculty of Medicine, University of Oslo, Campus at Akershus University Hospital, Norway; 36 Cancer Epidemiology Division, Cancer Council Victoria, Melbourne, Victoria, Australia; 37 Centre for Epidemiology and Biostatistics, Melbourne School of Population and Global Health, The University of Melbourne, Melbourne, Victoria, Australia; 38 Precision Medicine, School of Clinical Sciences at Monash Health, Monash University, Clayton, Victoria, Australia; 39 Paris-Saclay University, UVSQ, Gustave Roussy, Inserm, CESP, Villejuif, France; 40 Department of Cancer Genetics, Therapeutics and Ultrastructural Pathology, The Cyprus Institute of Neurology & Genetics, Nicosia, Cyprus; 41 Cyprus School of Molecular Medicine, The Cyprus Institute of Neurology & Genetics, Nicosia, Cyprus; 42 Center for Familial Breast and Ovarian Cancer, Faculty of Medicine and University Hospital Cologne, University of Cologne, Cologne, Germany; 43 Center for Integrated Oncology, Faculty of Medicine and University Hospital Cologne, University of Cologne, Cologne, Germany; 44 Department of Oncology, Södersjukhuset, Stockholm, Sweden; 45 Molecular Genetics of Breast Cancer, German Cancer Research Center, Heidelberg, Germany; 46 Translational Cancer Research Area, University of Eastern Finland, Kuopio, Finland; 47 Institute of Clinical Medicine, Pathology and Forensic Medicine, University of Eastern Finland, Kuopio, Finland; 48 Saw Swee Hock School of Public Health, National University of Singapore and National University Health System, Singapore, Singapore; 49 Department of Medicine, Yong Loo Lin School of Medicine, National University of Singapore and National University Health System, Singapore, Singapore; 50 Department of Surgery, National University Health System, Singapore, Singapore; 51 Dr. Margarete Fischer-Bosch-Institute of Clinical Pharmacology, Stuttgart, Germany; 52 University of Tübingen, Tübingen, Germany; 53 Division of Cancer Sciences, University of Manchester, Manchester, England; 54 Department of Genetics and Pathology, Pomeranian Medical University, Szczecin, Poland; 55 Independent Laboratory of Molecular Biology and Genetic Diagnostics, Pomeranian Medical University, Szczecin, Poland; 56 Department of Genetics and Fundamental Medicine, Bashkir State University, Ufa, Russia; 57 Department of Medical Genetics, Oslo University Hospital and University of Oslo, Oslo, Norway; 58 Institute of Clinical Medicine, Faculty of Medicine, University of Oslo, Oslo, Norway; 59 Human Genetics Division, Genome Institute of Singapore, Singapore, Singapore; 60 Breast Department, KK Women’s and Children’s Hospital, Singapore, Singapore; 61 SingHealth Duke-NUS Breast Centre, Singapore, Singapore; 62 Department of Molecular Medicine and Surgery, Karolinska Institutet, Stockholm, Sweden; 63 Department of Clinical Genetics, Karolinska University Hospital, Stockholm, Sweden; 64 Division of Population Health, Health Services Research and Primary Care, School of Health Sciences, Faculty of Biology, Medicine and Health, University of Manchester, Manchester, England; 65 Biobank of Eastern Finland, Kuopio University Hospital, Kuopio, Finland; 66 Department of Clinical Science and Education, Södersjukhuset, Karolinska Institutet, Stockholm, Sweden; 67 Department of Medical Oncology, University Hospital of Heraklion, Heraklion, Greece; 68 Department of Surgery, Faculty of Medicine University of Malaya, UM Cancer Research Institute, Kuala Lumpur, Malaysia; 69 Institute for Medical Biometry and Epidemiology, University Medical Center Hamburg-Eppendorf, Hamburg, Germany; 70 Centro de Investigación en Red de Enfermedades Raras, Madrid, Spain; 71 Genome Diagnostics Program, IFOM–the FIRC Institute of Molecular Oncology, Milan, Italy; 72 Unit of Molecular Bases of Genetic Risk and Genetic Testing, Department of Research, Fondazione IRCCS Istituto Nazionale dei Tumori, Milan, Italy; 73 Department of Oncology, University Hospital of Larissa, Larissa, Greece; 74 School of Cancer & Pharmaceutical Sciences, Comprehensive Cancer Centre, Guy’s Campus, King’s College London, London, England; 75 Center for Molecular Medicine Cologne, Faculty of Medicine and University Hospital Cologne, University of Cologne, Cologne, Germany; 76 Centre for Cancer Genetic Epidemiology, Department of Oncology, University of Cambridge, Cambridge, England; 77 Department of Clinical Pathology, University of Melbourne, Melbourne, Victoria, Australia; 78 Research Department, Peter MacCallum Cancer Center, Melbourne, Victoria, Australia; 79 Sir Peter MacCallum Department of Oncology, University of Melbourne, Melbourne, Victoria, Australia; 80 Institute of Cancer and Genomic Sciences, University of Birmingham, Birmingham, England; 81 Wellcome Trust Centre for Human Genetics and Oxford National Institute for Health Research Biomedical Research Centre, University of Oxford, Oxford, England; 82 Institute of Human Genetics, Pontificia Universidad Javeriana, Bogota, Colombia; 83 Subang Jaya Medical Centre, Subang Jaya, Selangor, Malaysia; 84 Department of Human Genetics, Leiden University Medical Center, Leiden, the Netherlands; 85 Division of Oncology and Pathology, Department of Clinical Sciences Lund, Lund University, Lund, Sweden; 86 Department of Obstetrics and Gynecology, Helsinki University Hospital, University of Helsinki, Helsinki, Finland; 87 Breast Cancer Research Programme, Cancer Research Malaysia, Subang Jaya, Selangor, Malaysia; 88 Department of Pathology, Leiden University Medical Center, Leiden, the Netherlands; 89 Department of Clinical Genetics, Leiden University Medical Center, Leiden, the Netherlands

## Abstract

**Importance:**

Rare germline genetic variants in several genes are associated with increased breast cancer (BC) risk, but their precise contributions to different disease subtypes are unclear. This information is relevant to guidelines for gene panel testing and risk prediction.

**Objective:**

To characterize tumors associated with BC susceptibility genes in large-scale population- or hospital-based studies.

**Design, Setting, and Participants:**

The multicenter, international case-control analysis of the BRIDGES study included 42 680 patients and 46 387 control participants, comprising women aged 18 to 79 years who were sampled independently of family history from 38 studies. Studies were conducted between 1991 and 2016. Sequencing and analysis took place between 2016 and 2021.

**Exposures:**

Protein-truncating variants and likely pathogenic missense variants in *ATM*, *BARD1*, *BRCA1*, *BRCA2*, *CHEK2*, *PALB2*, *RAD51C*, *RAD51D*, and *TP53*.

**Main Outcomes and Measures:**

The intrinsic-like BC subtypes as defined by estrogen receptor, progesterone receptor, and ERBB2 (formerly known as *HER2*) status, and tumor grade; morphology; size; stage; lymph node involvement; subtype-specific odds ratios (ORs) for carrying protein-truncating variants and pathogenic missense variants in the 9 BC susceptibility genes.

**Results:**

The mean (SD) ages at interview (control participants) and diagnosis (cases) were 55.1 (11.9) and 55.8 (10.6) years, respectively; all participants were of European or East Asian ethnicity. There was substantial heterogeneity in the distribution of intrinsic subtypes by gene. *RAD51C*, *RAD51D*, and *BARD1* variants were associated mainly with triple-negative disease (OR, 6.19 [95% CI, 3.17-12.12]; OR, 6.19 [95% CI, 2.99-12.79]; and OR, 10.05 [95% CI, 5.27-19.19], respectively). *CHEK2* variants were associated with all subtypes (with ORs ranging from 2.21-3.17) except for triple-negative disease. For *ATM* variants, the association was strongest for the hormone receptor (HR)^+^ERBB2^−^ high-grade subtype (OR, 4.99; 95% CI, 3.68-6.76). *BRCA1* was associated with increased risk of all subtypes, but the ORs varied widely, being highest for triple-negative disease (OR, 55.32; 95% CI, 40.51-75.55). *BRCA2* and *PALB2* variants were also associated with triple-negative disease. *TP53* variants were most strongly associated with HR^+^ERBB2^+^ and HR^–^ERBB2^+^ subtypes. Tumors occurring in pathogenic variant carriers were of higher grade. For most genes and subtypes, a decline in ORs was observed with increasing age. Together, the 9 genes were associated with 27.3% of all triple-negative tumors in women 40 years or younger.

**Conclusions and Relevance:**

The results of this case-control study suggest that variants in the 9 BC risk genes differ substantially in their associated pathology but are generally associated with triple-negative and/or high-grade disease. Knowing the age and tumor subtype distributions associated with individual BC genes can potentially aid guidelines for gene panel testing, risk prediction, and variant classification and guide targeted screening strategies.

## Key Points

**Question:**

What breast tumor characteristics are associated with rare pathogenic protein truncating or missense variants in breast cancer susceptibility genes?

**Findings:**

In this case-control study involving 46 387 control participants and 42 680 women with a diagnosis of breast cancer, pathology features (eg, tumor subtype, morphology, size, TNM stage, and lymph node involvement) associated with rare germline (likely) pathogenic variants in 9 different breast cancer susceptibility genes were studied. Substantial differences in tumor subtype distribution by gene were found.

**Meaning:**

The results of this study suggest that tumor subtypes differ by gene; these findings can potentially inform guidelines for gene panel testing, risk prediction in unaffected individuals, variant classification, and understanding of breast cancer etiology.

## Teaser

This case-control study examines tumors associated with breast cancer susceptibility genes in large-scale population- or hospital-based studies.

## Introduction

Breast cancer (BC) is a heterogeneous disease; different subtypes are associated with distinct biology, prognosis, and potential for therapy.^1,2,3^ There is evidence that inherited genetic predisposition contributes to this heterogeneity.^4,5^ However, data for detailed analysis of tumor pathologies that are associated with most BC susceptibility genes have been limited, particularly in population-based studies. Recent results from 2 large-scale sequencing studies, BRIDGES^6^ and CARRIERS,^7^ found evidence of an association with BC risk for germline protein-truncating variants (PTVs) and/or rare missense variants (MSVs) in 9 genes: *ATM, BARD1*, *BRCA1*, *BRCA2*, *CHEK2, PALB2, RAD51C*, *RAD51D*, and *TP53.* Women carrying variants in these genes may be offered enhanced screening, including by magnetic resonance imaging, risk-reducing surgery, chemoprevention, and genetic counselling; knowledge of germline gene variants also affects treatment.^8^ Intrinsic BC subtypes have been defined on the basis of patterns of gene expression; these include luminal-A, which defines a subset of hormone receptor–positive tumors that are associated with a good 5-year prognosis, and luminal-B, ERBB2-enriched and basal tumors with poorer prognosis.^2,9^ Gene expression data are not routinely available in diagnostic laboratories, but large-scale epidemiological studies can use subtypes based on immunochemical markers to define intrinsic-like surrogates that are broadly associated with the molecular subtypes.^10,11^ In this article, we use data from BRIDGES to assess associations between variants in these genes and pathological features of nonmetastasized breast tumors relevant to prognosis and/or distinct therapeutic options. We further quantify the contribution of rare BC susceptibility genes to the development of distinct BC subtypes in women of different ages.

## Methods

### Studies and Inclusion Criteria

The BRIDGES study included women with BC and unaffected control participants who were participating in the Breast Cancer Association Consortium (https://bcac.ccge.medschl.cam.ac.uk/; eTable 1 in Supplement 1). The analyses presented in this article are based on cases from the subset of population-based or hospital-based studies that were sampled independently of family history, together with population-matched control participants (38 studies). Women aged between 18 and 79 years were included. Pathology information from the first primary invasive BC was considered. Cases in which the index tumor was the second tumor and patients with metastases at initial diagnosis were excluded.^12^ All studies were approved by the relevant ethical review boards, and participants provided written informed consent.

### Laboratory Methods, Variant Calling, and Classification

We focused on 9 genes with evidence of an association with BC.^6^ We considered PTVs for all 9 genes, and rare (carrier frequency <0.1%) MSVs in *BRCA1*, *BRCA2* and *TP53* that were likely pathogenic according to adaptations of the American College of Medical Genetics guidelines.^6^ Approximately 80% of *CHEK2* PTVs were c.1100delC. The *TP53* PTV and MSV carriers were considered together. Carriers of *BRCA1* and *BRCA2* PTVs were excluded from the analyses of other genes. Carriers of PTVs in *BRCA1* and *BRCA2* and women who harbored a pathogenic variant in more than 1 non-*BRCA* gene were also excluded. *Noncarriers* were defined as women without PTVs or MSVs in any of the genes. Further details are provided in the eMethods in Supplement 1.

### Tumor Pathology

Pathology information was based on histology and immunohistochemistry results from medical records, rescored whole slides, or tumor microarrays that were curated in the Breast Cancer Association Consortium database, version 12.^13,14^ Markers included estrogen receptor (ER), progesterone receptor (PR), and erb-b2 receptor tyrosine kinase 2 (ERBB2, formerly known as HER2) status, which was denoted as positive or negative; histological grade (grades 1, 2, and 3); morphology; tumor size (<2, 2-5, or >5 cm); lymph node involvement (yes/no); and TNM stage (I, II, and III). For the purposes of this analysis, we defined 5 clinically relevant intrinsic subtypes based on available immunohistochemistry and grade: HR^+^ERBB2^−^ low (/intermediate) grade, HR^+^ERBB2^+^, and HR^+^ERBB2^−^ high grade, HR^−^ERBB2^+^ and triple negative (TN). Grades 1 and 2 were considered low-grade and grade 3 high-grade disease (eTable 2 in Supplement 1).^11,12,15,16^

### Statistical Analysis

Analyses were based on estimating the odds ratios (ORs) associated with carrying any PTV (or pathogenic MSV) in each gene. First, complete-case analyses based on all available data were conducted. Case-control analyses were used to estimate the OR for developing a tumor of a particular subtype according to single markers and case-only analyses to evaluate the evidence for differences by subtype. Logistic regression was used for binary characteristics and multinomial logistic regression for multicategory tumor characteristics. For multicategory outcomes, a model in which the log(OR) varied linearly with the outcome level was also fitted. Analyses were adjusted for age (defined as age at diagnosis for patients and age at interview for control participants) and country of origin of the study.

To evaluate heterogeneity of risk by intrinsic tumor subtypes, we first imputed missing pathology variables using Multiple Imputation by Chained Equations. Intrinsic subtypes were constructed for each of 100 imputed data sets, and the results of multinomial regression for each imputed data set were pooled. We also compared these data with results obtained after imputing tumor pathology using an expectation-maximization (EM) algorithm (eMethods in Supplement 1).^17^ We investigated interactions with age for each gene according to tumor subtype by including an age x variant product term in the model and also estimated the proportion of BC cases, by age-group and intrinsic subtype, for pathogenic variants in each gene.

Associations between (likely) pathogenic variant carrier status and tumor size and lymph node status were evaluated. Analyses were also conducted that included size, lymph node status, and intrinsic subtype in the same model and PR status and the HR-positive subtypes in the same model.

Gene-specific cumulative risks for each subtype were calculated by combining age-specific OR estimates with 2016 UK population incidence rates as a baseline and accounting for competing risk of not developing BC of a different subtype. Age-specific and gene-specific subtype proportions for tumor subtypes included in the risk prediction algorithm BOADICEA^18^ were also calculated (eMethods in Supplement 1).

Analyses were conducted using RStudio, version 1.2.5033 (RStudio); Stata, version 14.2 (StataCorp); and GFortran. Statistical significance was set at *P* < .05.

## Results

### Study Characteristics

The study comprised 46 387 control participants and 42 680 women with a diagnosis of BC from 22 countries, with mean (SD) ages at interview and diagnosis of 55.1 (11.9) and 55.8 (10.6) years, respectively (eTable 3 in Supplement 1). Numbers of variant carriers by gene are shown in eTable 4 in Supplement 1 and patterns of missingness in pathology data in eTable 5 in Supplement 1 and eTable 1 in Supplement 2; for ER status, 18%; grade, 18%; PR status, 32%; and ERBB2 status, 43% of data were missing. There was no association between missingness and genotype.

Single marker analyses were based on complete data (eFigure 1 in Supplement 1 and eTables 2 and 3 in Supplement 2). The remaining analyses were carried out following imputation of missing data.

### Distribution of Intrinsic Tumor Subtypes and Age Trends

**Figure 1.**
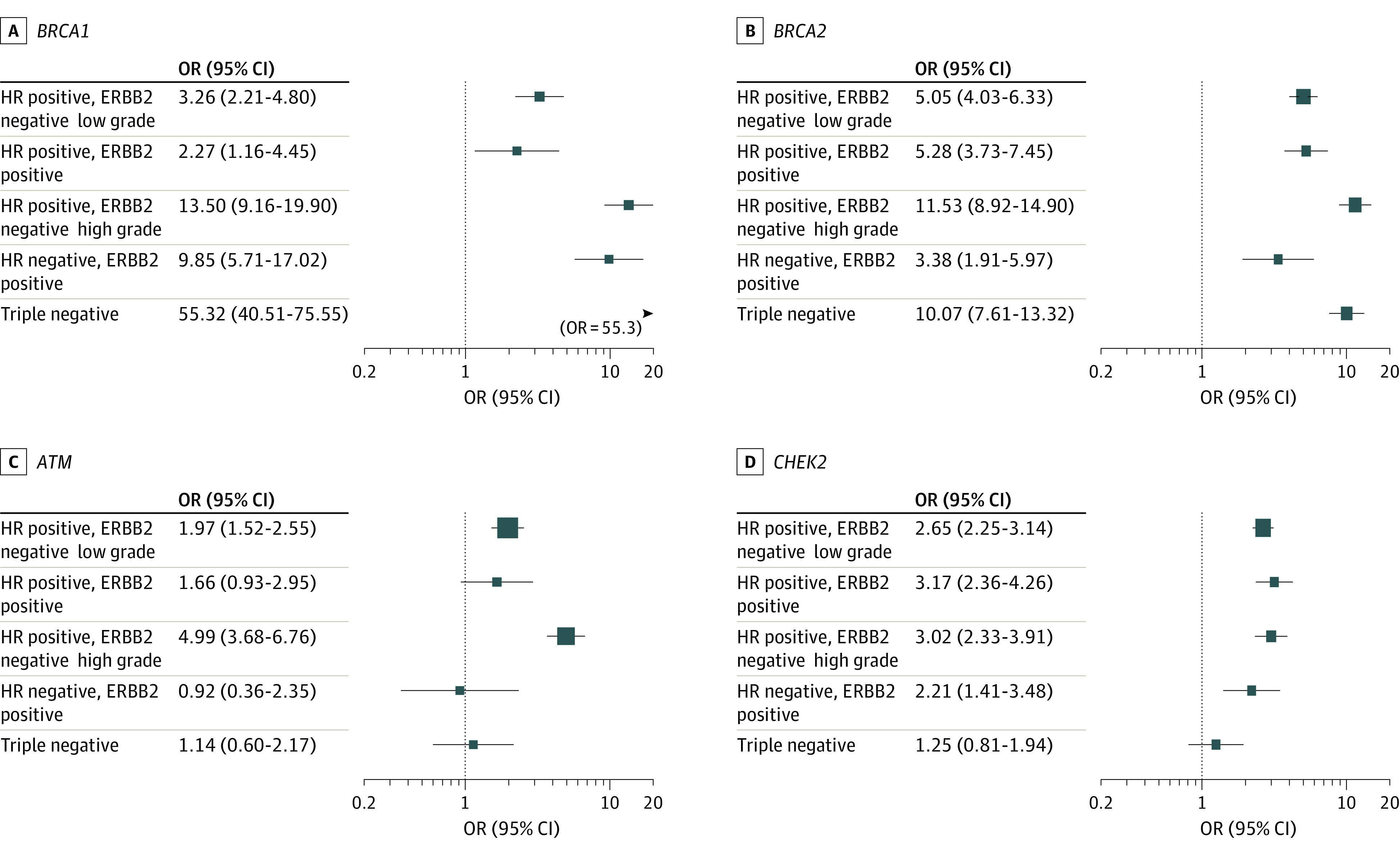
Association Odds Ratios (ORs) for Protein-Truncating Variant Carrier Status in Breast Cancer Susceptibility Genes *BRCA1*, *BRCA2*, *ATM*, and *CHEK2* and Intrinsic Subtypes of Breast Cancer Multiple Imputation by Chained Equations imputation was conducted as described in the Methods and intrinsic subtypes constructed for each imputed data set. Multinomial logistic regression was conducted with intrinsic subtypes as the outcome variable, adjusting by age at diagnosis/interview and country, and the results of these analyses were pooled. These results are also shown in eTable 4 in Supplement 2. HR indicates hormone receptor.

**Figure 2.**
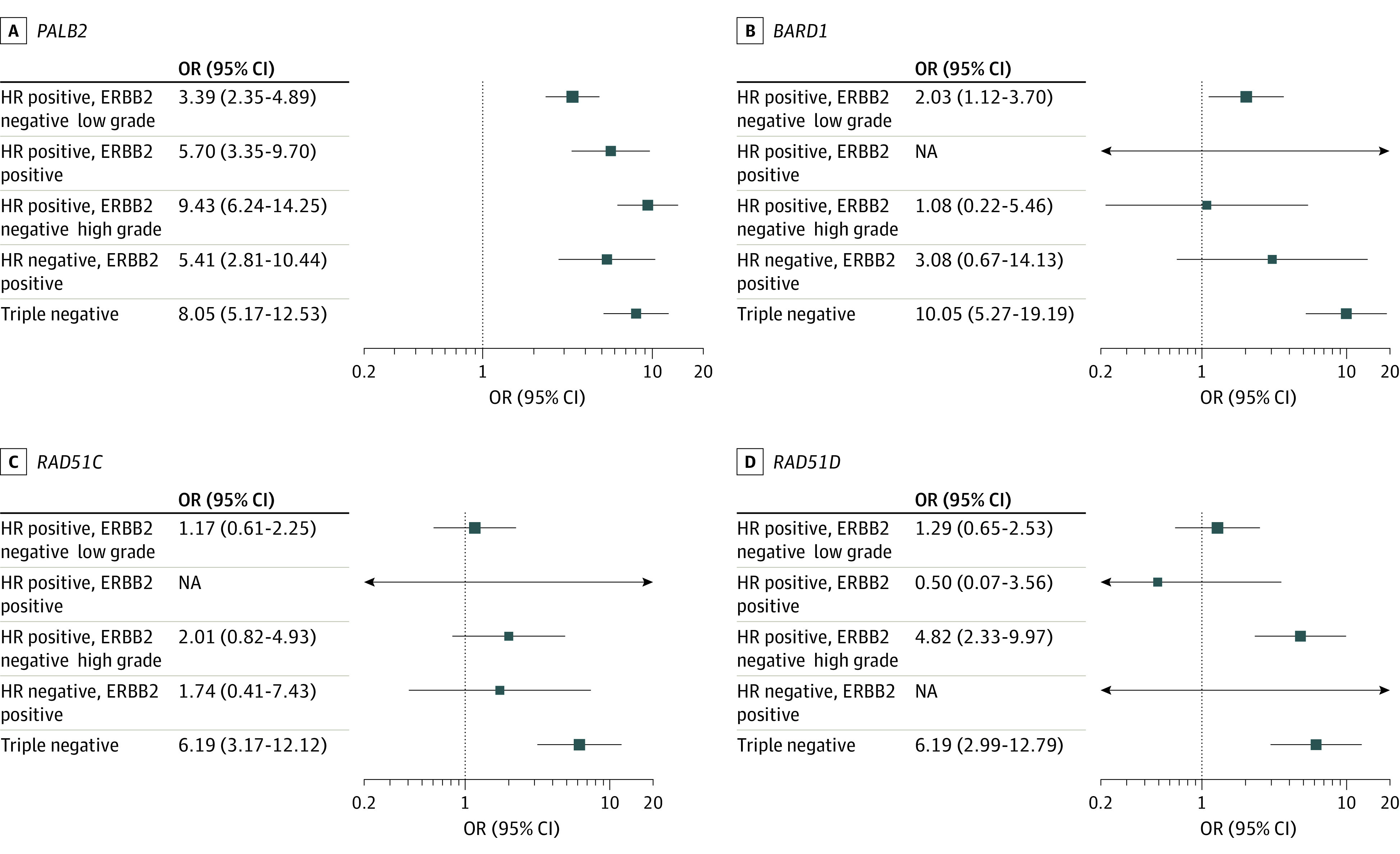
Association Odds Ratios (ORs) for Protein-Truncating Variant Carrier Status in Breast Cancer Susceptibility Genes *PALB2*, *BARD1*, *RAD51C*, and *RAD51D* and Intrinsic Subtypes of Breast Cancer Multiple Imputation by Chained Equations imputation was conducted as described in the Methods and intrinsic subtypes constructed for each imputed data set. Multinomial logistic regression was conducted with intrinsic subtypes as the outcome variable, adjusting by age at diagnosis/interview and country, and the results of these analyses were pooled. These results are also shown in eTable 4 in Supplement 2. HR indicates hormone receptor; NA, not applicable.

The PTVs in all 9 BC genes showed evidence of variation in the ORs among the 5 intrinsic subtypes (Figure 1, Figure 2; eFigures 2-5 and eTables 6 and 7 in Supplement 1; eTable 4 in Supplement 2). For *BRCA1* PTV carriers, the OR was highest (OR, 55.32; 95% CI, 40.51-75.55) for TN disease, much lower for HR^+^ERBB2^−^ low-grade disease (OR, 3.26; 95% CI, 2.21-4.80) and HR^+^ERBB2^+^ disease (OR, 2.27; 95% CI, 1.16-4.45), and intermediate for HR^+^ERBB2^−^ high-grade and HR^−^ERBB2^+^ disease (OR, 13.5 [95% CI, 9.16-19.90] and OR, 9.85 [95% CI, 5.71-17.02], respectively). Associations between *BRCA2* PTVs and intrinsic subtypes were more homogeneous across subtypes, with higher ORs associated with HR^+^ERBB2^−^ high-grade disease (OR, 11.53; 95% CI, 8.92-14.90) and TN tumors (OR, 10.07; 95% CI, 7.61-13.32). For *ATM*, the association was strongest for HR^+^ERBB2^−^ high-grade tumors (OR, 4.99; 95% CI, 3.68-6.76). *CHEK2* PTVs were associated with similar ORs with all subtypes except TN, for which there was no evidence of association. *PALB2* PTVs were associated with all subtypes, but with higher ORs for HR^+^ERBB2^−^ high-grade (OR, 9.43; 95% CI, 6.24-14.25) and TN disease (OR, 8.05; 95% CI, 5.17-12.53).

The PTVs in *RAD51C*, *RAD51D*, and *BARD1* were most strongly associated with TN disease (OR, 6.19 [95% CI, 3.17-12.12]; OR, 6.19 [95% CI, 2.99-12.79]; and OR, 10.05 [95% CI, 5.27-19.19], respectively). *RAD51D* PTVs were also associated with HR^+^ERBB2^−^ high-grade tumors.

Similar to PTVs, carriers of *BRCA1* MSV were strongly enriched for TN disease (eTable 4 in Supplement 2 and eFigure 6 in Supplement 1). The ORs were higher than those for PTVs for all of the intrinsic subtypes, although these differences were not statistically significant. For *BRCA2*, the ORs for MSVs and PTVs were similar. *TP53* variants were associated with HR^−^ERBB2^+^ and HR^+^ERBB2^+^ but not TN disease.

A decline in the ORs with increasing age was observed for *BRCA1* and *BRCA2*; this trend was similar for all subtypes (OR, 0.96 per year for both genes; *P* = 7.05 × 10^−7^ and 3.14 × 10^−11^ for *BRCA1* [95% CI, 0.94-0.98] and *BRCA2* [95% CI, 0.95-0.97], respectively; eTable 4 in Supplement 2). The ORs also declined with age for *CHEK2*, but the trend was much weaker. There was no evidence of a decline in the ORs for *ATM*, *BARD1*, *RAD51C*, or *RAD51D*, but the confidence limits for the last 3 genes were wide.

We further stratified HR-positive subtypes by PR expression to determine whether carrier status was associated with PR. For *BRCA1*, the ORs were lowest for ER^+^, PR^+^ tumors compared with other categories (eTable 8 in Supplement 1). Consistent with this observation, *BRCA1* PTV carriers were more likely to be PR negative, even after adjusting for intrinsic subtype. There was also some weak evidence for *BRCA2* PTVs and PR negativity, but no evidence for the other genes.

### Association Between Breast Cancer Susceptibility Genes and Other Prognostic Factors

The PTVs in *BRCA2*, *CHEK2*, and *PALB2* were associated with larger tumor size, lymph node involvement, and higher stage at diagnosis (eFigure 1 in Supplement 1). The individual associations with larger tumor size and lymph node involvement remained significant after adjusting for intrinsic subtypes. The association between PTVs in all 9 genes with intrinsic subtypes remained similar after including size and lymph node status in the model (eTable 5 in Supplement 2).

For each gene, most BCs were carcinoma no special type (ductal carcinoma); in aggregate, 71% of tumors in carriers and 68% in noncarriers were ductal carcinoma. *BRCA1* tumors were less likely to be lobular than ductal (OR, 0.40; 95% CI, 0.25-0.63) but more likely to be medullary than nonmedullary (OR, 5.24; 95% CI, 3.34-8.22) (eTables 2 and 3 in Supplement 2). *TP53* tumors were more likely to be mixed lobular and ductal than ductal carcinoma (OR, 7.01; 95% CI, 3.04-16.17; *P* = 5 × 10^−6^). Otherwise, tumors associated with variations in the other BC genes were not enriched for any particular morphology.

### Prevalence of Pathogenic Variants According to Subtypes and Age

We assessed the association between rare variants in BC susceptibility genes with the burden of disease in women of different ages (eFigures 7-12 and eTable 7 in Supplement 1). Together, the 9 genes were associated with 14.4% of all tumors in women 40 years or younger but less than 4% in women older than 60 years. Among younger women, the prevalence of variants combined was higher among women with TN and HR^+^ERBB2^−^ high-grade tumors than those with other subtypes. The highest prevalence (27.3%) was among women 40 years or younger with TN tumors, mainly driven by *BRCA1* (eFigure 7 in Supplement 1). The combined prevalence of pathogenic variants was close to or exceeded 10% for all subtypes in women younger than 40 years and for TN and HR^+^ERBB2^−^ high-grade disease in women aged 40 to 59 years. Although *TP53*-related tumors comprised only a small proportion of ERBB2-positive disease, approximately 70% of *TP53* tumors among women 40 years or younger were ERBB2-positive.

### Age-Specific Cumulative Risk of Developing Intrinsic BC Tumor Subtypes

**Figure 3.**
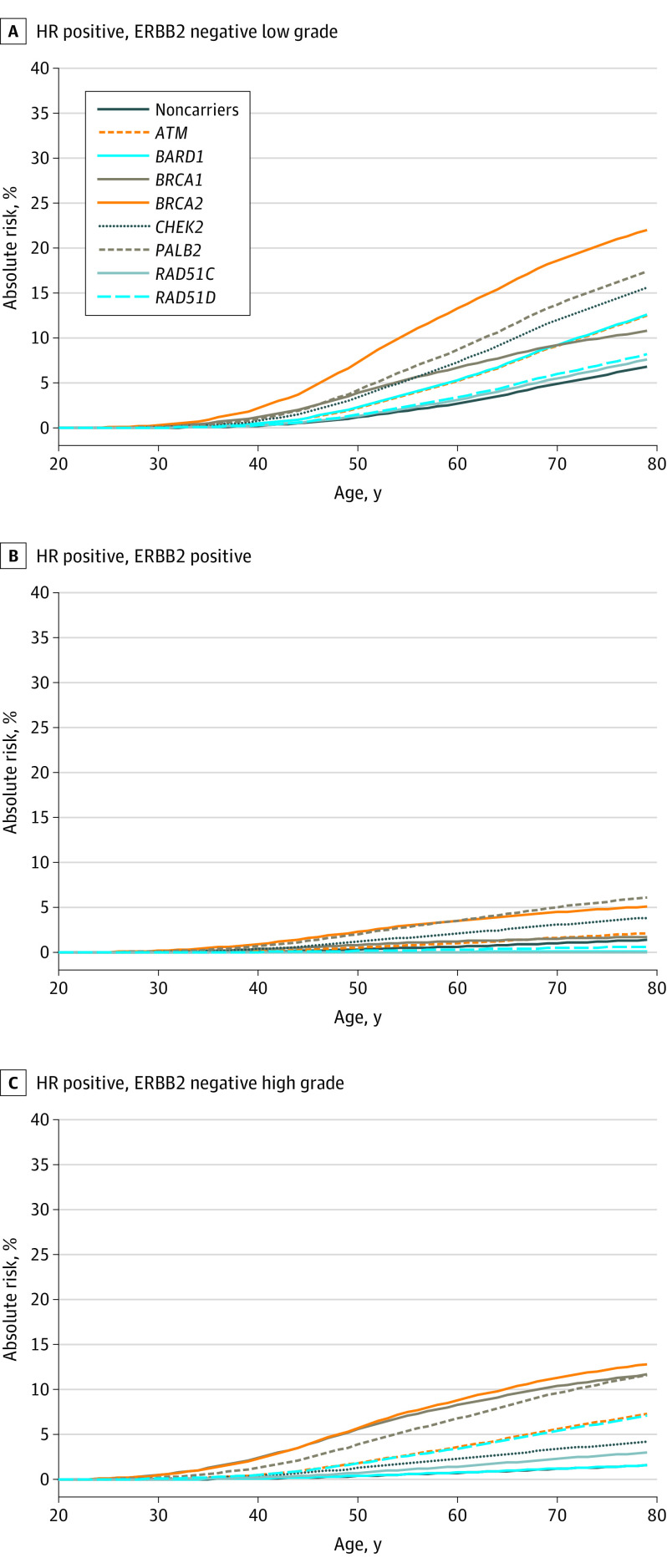
Estimates of Cumulative Risks of Breast Cancer by Age and Hormone Receptor (HR)–Positive Subtype for Protein-Truncating Variants in 8 Breast Cancer Susceptibility Genes Age-, gene-, and subtype-specific cumulative risks were calculated as described in the Methods and eMethods in Supplement 1. Baseline incidence rates were derived from UK breast cancer incidence rates for 2016 (https://www.cancerresearchuk.org/health-professional/cancer-statistics/statistics-by-cancer-type/breast-cancer/incidence-invasive).

**Figure 4.**
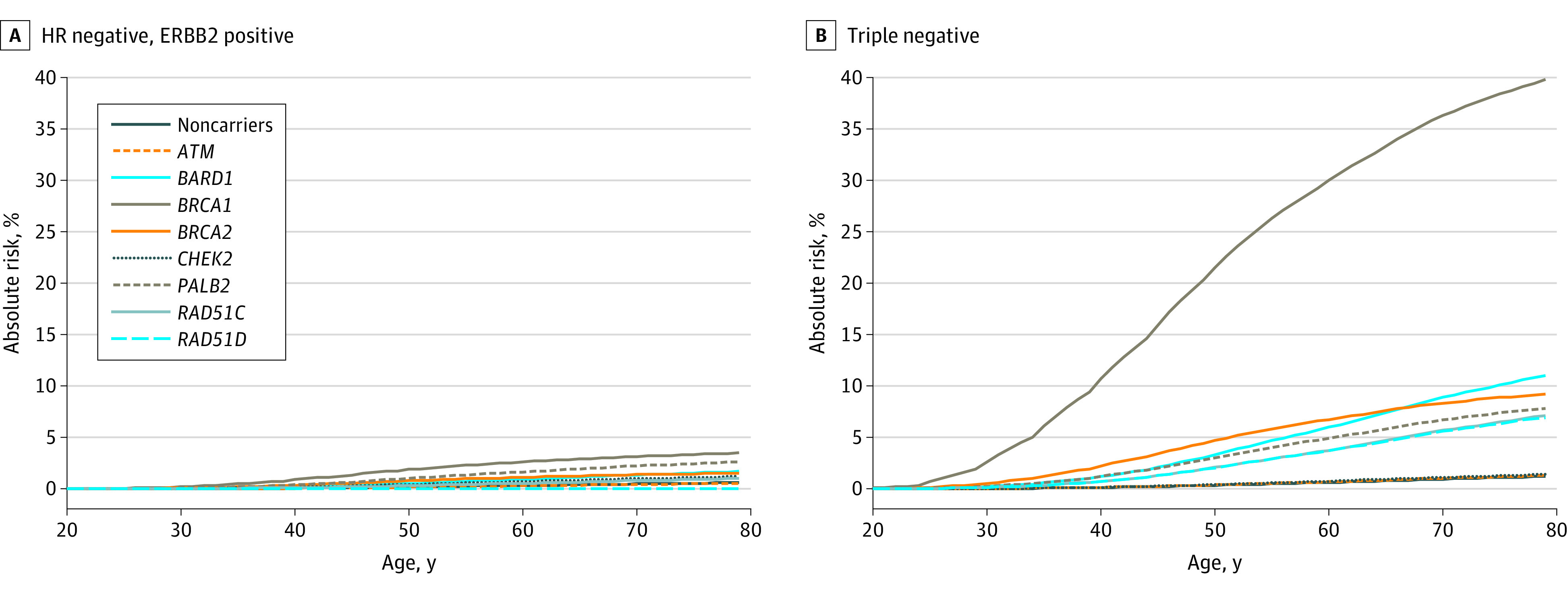
Estimates of Cumulative Risks of Breast Cancer by Age and Hormone Receptor (HR)–Negative Subtype for Protein-Truncating Variants in 8 Breast Cancer Susceptibility Genes Age-, gene-, and subtype-specific cumulative risks were calculated as described in the Methods and eMethods in Supplement 1. Baseline incidence rates were derived from UK breast cancer incidence rates for 2016 (https://www.cancerresearchuk.org/health-professional/cancer-statistics/statistics-by-cancer-type/breast-cancer/incidence-invasive).

Estimated cumulative risks according to intrinsic subtypes are shown in Figure 3 and Figure 4. The estimated risk for TN tumors was highest for *BRCA1* (40% by age 80 years), and 7% to 12% for *BRCA2, BARD1, PALB2, RAD51C*, and *RAD51D*. In contrast, the highest risks for HR^+^ERBB2^−^ low-grade disease were associated with *BRCA2* (22%) followed by *PALB2* and *CHEK2*.

## Discussion

This case-control study evaluated the pathology of BCs developing in carriers of PTVs and/or rare MSVs in 9 BC susceptibility genes: *ATM, BARD1*, *BRCA1*, *BRCA2*, *CHEK2, PALB2, RAD51C*, *RAD51D*, and *TP53* in a large multicenter collaborative study comprising population-based and hospital-based studies. The pattern of intrinsic subtypes and markers of tumor aggressiveness differed between carriers of variants in individual BC susceptibility genes and noncarriers. As expected,^19,20^ BC in *BRCA1* carriers were strongly enriched for TN tumors, with TN disease representing approximately 60% of all tumors and approximately 70% of tumors in women 40 years and younger. However, the risks for all other subtypes were also increased (ORs, 2.27-13.5). For *BRCA2*, the highest ORs were for HR^+^ERBB2^−^ high-grade and TN disease, which was consistent with the strong association with ERBB2 negativity.^21,22^ The most common subtype (43% of cases) was HR^+^ERBB2^−^ low (/intermediate)–grade disease, but a clear excess of TN disease (approximately 18% of tumors) was apparent, even at younger ages. Subtype-specific associations for *BRCA1* and *BRCA2* MSVs were similar to those for PTVs in the corresponding genes.

Although the ORs were lower, the pattern of intrinsic subtypes for *PALB2* carriers was very similar to that for *BRCA2* carriers (Figures 1 and 2), with variation in both genes being associated with ERBB2 negativity and TN disease.^23^ This similarity may reflect the closely associated functions of *PALB2* and *BRCA2* in the DNA damage response.^24^

Conversely, the profile of intrinsic subtypes associated with *BARD1* carriers was similar to *BRCA1* carriers, with an excess of TN tumors (40% of cases), albeit the overall risk was much lower. Consistent with this observation, *Bard1* and *Brca1* knockout mice have similar phenotypes.^25^ *BARD1* and *BRCA1* proteins form a stable complex, the heterodimer coordinating a range of cellular pathways to maintain genomic stability. Although *BRCA1* requires *BARD1* for stability and tumor suppressor functions, *BARD1* also plays distinct roles in cell cycle progression.^25,26^

Carriers of PTVs in *ATM* and *CHEK2* were more strongly associated with ER-positive disease, but this study highlights some differences. For *ATM*, the association was particularly strong for HR^+^ERBB2^−^ high-grade tumors (OR, 4.99; 95% CI, 3.68-6.76), with weaker associations for the other HR-positive subtypes (although HR^+^ERBB2^−^ low-grade tumors were still the most common). An association with the luminal B subtype has been reported previously in a small data set (n = 28).^27^ *CHEK2* was associated with a similar OR for all the HR-positive subtypes and increased risk of HR^−^ERBB2^+^, but not TN disease. *ATM* plays a central role in the activation of DNA damage response and cell cycle checkpoint control, while *CHEK2* is involved downstream of *ATM* in cell cycle arrest, apoptosis, and DNA repair.^28,29^

*RAD51C* and *RAD51D* are known ovarian cancer susceptibility genes and are more recently associated with BC,^6,30^ in particular with TN disease.^4,31,32^ In the present study, ORs for TN disease for PTVs in both genes were approximately 6.0. The subtype distribution of *RAD51C* is similar to *RAD51D*, reflecting their closely associated functions. We did observe an excess of HR^+^ERBB2^−^ high-grade tumors in *RAD51D* but not *RAD51C* carriers; however, the numbers of PTV carriers were small and we cannot exclude the subtype distributions being similar.

*TP53* tumors were strongly enriched for ERBB2-positive subtypes (46% of cases), which was consistent with earlier studies in patients with or without Li-Fraumeni syndrome^33,34^ and examination of patients identified by multigene panel testing.^35^ We also observed an association with mixed ductal and lobular morphology, tumors that comprise distinct but clonally related morphological components.^36^

Pathogenic PTVs and MSVs in these 9 BC susceptibility genes were disproportionately associated with more aggressive BC, particularly among younger women. Carriers of rare genetic variants in the 9 genes constituted almost a third of women who received a diagnosis at or younger than 40 years of TN disease and approximately 16% of women with HR^+^ERBB2^−^ high-grade disease. All genes except *CHEK2* were more strongly associated with high-grade disease. Across genes, 27% to 72% of tumors were grade 3 (eTable 2 in Supplement 2). Previously studies have suggested that tumors in carriers of rare PTVs are larger^23,37,38,39,40^ and more likely to be identified as interval rather than screen-detected cancers.^37^ In the present study, *BRCA2*-, *CHEK2*-, and *PALB2*-associated tumors were larger and more likely to be lymph node positive.

Despite the strong enrichment of TN disease for many of the genes, most carriers will still develop HR^+^ disease. With the exception of *BRCA1*, the most common subtype for all genes was HR^+^ERBB2^−^ low (/intermediate)–grade disease (Figures 3 and 4). However, these absolute risk projections indicate average subtype-specific risks, while individual risk prediction should also consider polygenic modifiers, family history, and lifestyle and reproductive factors, as well as the risk of developing cancers at other sites.^41^ The age- and subtype-specific risk estimates (eTable 6 in Supplement 2 and eFigure 13 in Supplement 1) may be used to refine BC risk prediction algorithms, such as BOADICEA.^41^

These results may also inform guidelines for eligibility for gene panel sequencing and BC surveillance in the general population. The combined prevalence of pathogenic variants in any of the 9 genes reached 10% for TN cases in those who received a diagnosis when younger than 60 years and HR^+^ERBB2^−^ high-grade and HR^+^ERBB2^+^ cases in those who received a diagnosis at 40 years or younger (in HR^−^ERBB2^+^ cases, the prevalence was 9.4%). These are slight underestimates of the true frequency because some variants deleterious to gene function, notably large gene rearrangements, will have been missed in the targeted sequencing.^6^

Tumor characteristics can also be used in determining whether variants of uncertain significance are likely to be pathogenic based on the assumption that the tumor characteristics of pathogenic variants of uncertain significance will be similar to known pathogenic variants.^42^ Therefore, these data should improve the precision of variant classification algorithms and extend them to a larger set of genes.

### Strengths and Limitations

The strengths of this study are its large sample size (42 680 cases and 46 387 control participants) and sampling of cases independent of family history, while most earlier investigations have involved women who were ascertained in genetics clinics and selected based on family history, genotype, or pathology. The large sample size allowed us to obtain unbiased estimates of ORs and age interaction effects, while the sampling framework provided results that are particularly relevant as gene panel testing becomes applied at a general population level. Cases and control participants underwent sequencing on the same platform and using a single variant calling algorithm. We analyzed a comprehensive set of variables and their associations. Finally, the results found using 2 different imputation methods, Multiple Imputation by Chained Equations and an EM algorithm were consistent (eTable 9 in Supplement 1).

Despite the large size of this study, the sample size with complete pathology data was still limited for some genes. For example, ERBB2 status was missing for approximately 43% of samples, although missingness is likely to be random with respect to genotype, and imputation methods performed well. There was also minor heterogeneity in definition of stage, grade, and cutoffs for ER, PR, and ERBB2 across studies. The subtypes defined by immunohistochemical markers do not align perfectly with intrinsic subtypes defined by expression profiles, such as PAM-50,^43,44^ but such data are not available in large-scale epidemiological studies or routine practice. Finally, most participants were of European descent, and larger studies of women from other racial and ethnic groups will be important.

## Conclusions

This case-control study suggests that rare variants in BC susceptibility genes display marked heterogeneity with respect to tumor phenotype, but also similarities between genes that are consistent with known biological functions. This present study provides detailed quantification of subtype-specific BC risks; these can potentially improve risk prediction models and breast cancer prevention strategies.

## Notes

Supplement 1. eMethods. eTable 1. Description of studies included in this analysis eTable 2. Immunohistochemistry and tumor grade-based surrogates for five intrinsic breast cancer subtypes eTable 3. Numbers of cases and controls, and age distributions, by country of origin eTable 4. Numbers of variant carriers by breast cancer susceptibility gene eTable 5. Cross tabulation of ER, PR, HER2 and Grade data eTable 6. Distribution of intrinsic tumor subtypes in women of all ages and in different age groups, by breast cancer susceptibility gene. eTable 7. Prevalence of PTV and MSV in breast cancer susceptibility genes by intrinsic subtypes of breast cancer among women of different age groups at diagnosis eTable 8. Odds ratios for association between PTV and MSV carrier status and intrinsic subtypes refined by PR expression eTable 9. Odds ratios for association between PTV and MSV carrier status and intrinsic subtypes of breast cancer following imputation using an EM algorithm. eFigure 1. Case-only analysis of phenotypic markers and prognostic features by gene (complete case analysis) eFigure 2. Frequency histogram of intrinsic subtypes among noncarriers and carriers of PTVs and MSVs in 9 genes. eFigure 3. Frequency distribution of intrinsic subtypes among noncarriers and carriers of PTVs and MSVs in the 9 genes, in women aged ≤40 years. eFigure 4. Frequency distribution of intrinsic subtypes among noncarriers and carriers of PTVs and MSVs in the 9 genes, in women aged 41-60 years. eFigure 5. Frequency distribution of intrinsic subtypes among noncarriers and carriers of PTVs and MSVs in the 9 genes, in women aged >60 years. eFigure 6. Association odds ratios for MSV carrier status in BRCA1, BRCA2, TP53 and intrinsic subtypes of breast cancer. eFigure 7. Prevalence of PTV and MSV in breast cancer susceptibility genes by intrinsic subtypes of breast cancer among women aged ≤40 at diagnosis (A) HR+ HER2- low-grade (B) HR+ HER2- (C) HR+ HER2- high-grade (D) HR- HER2+ (E) TN. eFigure 8. Prevalence of PTV and MSV in breast cancer susceptibility genes by intrinsic subtypes of breast cancer among women aged 41-60 at diagnosis (A) HR+ HER2- low-grade (B) HR+ HER2- (C) HR+ HER2- high-grade (D) HR- HER2+ (E) TN. eFigure 9. Prevalence of PTV and MSV in breast cancer susceptibility genes by intrinsic subtypes of breast cancer among women aged >60 at diagnosis (A) HR+ HER2- low-grade (B) HR+ HER2- (C) HR+ HER2- high-grade (D) HR- HER2+ (E) TN. eFigure 10. Prevalence of PTV and MSV in breast cancer susceptibility genes by tumor grade among women aged ≤40 years at diagnosis with (A) grade 1 (B) grade 2 and (C) grade 3 breast cancer. eFigure 11. Prevalence of PTV and MSV in breast cancer susceptibility genes by tumor grade among women aged 41-60 years at diagnosis with (A) grade 1 (B) grade 2 and (C) grade 3 breast cancer. eFigure 12. Prevalence of PTV and MSV in breast cancer susceptibility genes by tumor grade among women aged >60 years at diagnosis with (A) grade 1 (B) grade 2 and (C) grade 3 breast cancer. eFigure 13. Smoothed proportions of subtypes used in BOADICEA for PTVs in ATM, BARD1, BRCA1, BRCA2, CHEK2, PALB2, RAD51C and RAD51D.

Supplement 2. eTable 1. Missing data by gene/mutation status and age at diagnosis eTable 2. Number of cases by subtype of breast cancer, as defined by individual tumor markers, and PTV/MSV carrier status for each gene eTable 3. Odds ratios for association between PTV and MSV carrier status and subtypes of breast cancer as defined by individual tumor markers (complete case analyses) eTable 4. Odds ratios for association between PTV and MSV carrier status and intrinsic subtypes of breast cancer following MICE imputation– analyses with and without an age interaction term eTable 5. Association between breast cancer genes and intrinsic subtypes - Joint models of intrinsic subtype, tumor size and lymph node involvement eTable 6. Smoothed proportions of subtypes used in BOADICEA for PTVs in ATM, BARD1, BRCA1, BRCA2 CHEK2, PALB2, RAD51C and RAD51D
