# Supplementary material for: Pathology of Tumors Associated With Pathogenic Germline Variants in 9 Breast Cancer Susceptibility Genes
Source: JAMA Oncol. 2022 Jan 27;8(3):e216744. doi: 10.1001/jamaoncol.2021.6744 (PMC8796069; doi:10.1001/jamaoncol.2021.6744)
Supplement: Supplement 1. — eMethods. eTable 1. Description of studies included in this analysis eTable 2. Immunohistochemistry and tumor grade-based surrogates for five intrinsic breast cancer subtypes eTable 3. Numbers of cases and controls, and age distributions, by country of origin eTable 4. Numbers of variant carriers by breast cancer susceptibility gene eTable 5. Cross tabulation of ER, PR, HER2 and Grade data eTable 6. Distribution of intrinsic tumor subtypes in women of all ages and in different age groups, by breast cancer susceptibility gene. eTable 7. Prevalence of PTV and MSV in breast cancer susceptibility genes by intrinsic subtypes of breast cancer among women of different age groups at diagnosis eTable 8. Odds ratios for association between PTV and MSV carrier status and intrinsic subtypes refined by PR expression eTable 9. Odds ratios for association between PTV and MSV carrier status and intrinsic subtypes of breast cancer following imputation using an EM algorithm. eFigure 1. Case-only analysis of phenotypic markers and prognostic features by gene (complete case analysis) eFigure 2. Frequency histogram of intrinsic subtypes among noncarriers and carriers of PTVs and MSVs in 9 genes. eFigure 3. Frequency distribution of intrinsic subtypes among noncarriers and carriers of PTVs and MSVs in the 9 genes, in women aged ≤40 years. eFigure 4. Frequency distribution of intrinsic subtypes among noncarriers and carriers of PTVs and MSVs in the 9 genes, in women aged 41-60 years. eFigure 5. Frequency distribution of intrinsic subtypes among noncarriers and carriers of PTVs and MSVs in the 9 genes, in women aged >60 years. eFigure 6. Association odds ratios for MSV carrier status in BRCA1, BRCA2, TP53 and intrinsic subtypes of breast cancer. eFigure 7. Prevalence of PTV and MSV in breast cancer susceptibility genes by intrinsic subtypes of breast cancer among women aged ≤40 at diagnosis (A) HR+ HER2- low-grade (B) HR+ HER2- (C) HR+ HER2- high-grade (D) HR- HER2+ (E) TN. eFigure 8. Prev [file jamaoncol-e216744-s001.pdf]

Supplemental Online Content

Breast Cancer Association Consortium. Pathology of tumors associated with pathogenic germline variants in 9 breast cancer susceptibility genes. *JAMA Oncol*. Published online January 27, 2022. doi:10.1001/jamaoncol.2021.6744

eMethods.
eTable 1. Description of studies included in this analysis
eTable 2. Immunohistochemistry and tumor grade-based surrogates for five intrinsic breast cancer subtypes
eTable 3. Numbers of cases and controls, and age distributions, by country of origin
eTable 4. Numbers of variant carriers by breast cancer susceptibility gene
eTable 5. Cross tabulation of ER, PR, HER2 and Grade data
eTable 6. Distribution of intrinsic tumor subtypes in women of all ages and in different age groups, by breast cancer susceptibility gene.
eTable 7. Prevalence of PTV and MSV in breast cancer susceptibility genes by intrinsic subtypes of breast cancer among women of different age groups at diagnosis
eTable 8. Odds ratios for association between PTV and MSV carrier status and intrinsic subtypes refined by PR expression
eTable 9. Odds ratios for association between PTV and MSV carrier status and intrinsic subtypes of breast cancer following imputation using an EM algorithm.
eFigure 1. Case-only analysis of phenotypic markers and prognostic features by gene (complete case analysis)
eFigure 2. Frequency histogram of intrinsic subtypes among noncarriers and carriers of PTVs and MSVs in 9 genes.
eFigure 3. Frequency distribution of intrinsic subtypes among noncarriers and carriers of PTVs and MSVs in the 9 genes, in women aged ≤40 years.
eFigure 4. Frequency distribution of intrinsic subtypes among noncarriers and carriers of PTVs and MSVs in the 9 genes, in women aged 41-60 years.
eFigure 5. Frequency distribution of intrinsic subtypes among noncarriers and carriers of PTVs and MSVs in the 9 genes, in women aged >60 years.
eFigure 6. Association odds ratios for MSV carrier status in *BRCA1*, *BRCA2*, *TP53* and intrinsic subtypes of breast cancer.
eFigure 7. Prevalence of PTV and MSV in breast cancer susceptibility genes by intrinsic subtypes of breast cancer among women aged ≤40 at diagnosis (A) HR+ HER2- low-grade (B) HR+ HER2- (C) HR+ HER2- high-grade (D) HR- HER2+ (E) TN.
eFigure 8. Prevalence of PTV and MSV in breast cancer susceptibility genes by intrinsic subtypes of breast cancer among women aged 41-60 at diagnosis (A) HR+ HER2- low-grade (B) HR+ HER2- (C) HR+ HER2- high-grade (D) HR- HER2+ (E) TN.
eFigure 9. Prevalence of PTV and MSV in breast cancer susceptibility genes by intrinsic subtypes of breast cancer among women aged >60 at diagnosis (A) HR+ HER2- low-grade (B) HR+ HER2- (C) HR+ HER2- high-grade (D) HR- HER2+ (E) TN.

© 2022 Breast Cancer Association Consortium. *JAMA Oncol.*

eFigure 10. Prevalence of PTV and MSV in breast cancer susceptibility genes by tumor grade among women aged ≤40 years at diagnosis with (A) grade 1 (B) grade 2 and (C) grade 3 breast cancer.
eFigure 11. Prevalence of PTV and MSV in breast cancer susceptibility genes by tumor grade among women aged 41-60 years at diagnosis with (A) grade 1 (B) grade 2 and (C) grade 3 breast cancer.
eFigure 12. Prevalence of PTV and MSV in breast cancer susceptibility genes by tumor grade among women aged >60 years at diagnosis with (A) grade 1 (B) grade 2 and (C) grade 3 breast cancer.
eFigure 13. Smoothed proportions of subtypes used in BOADICEA for PTVs in *ATM*, *BARD1*, *BRCA1*, *BRCA2*, *CHEK2*, *PALB2*, *RAD51C* and *RAD51D.*

This supplemental material has been provided by the authors to give readers additional information about their work.

eMethods

Studies and inclusion criteria

The BRIDGES study included samples from female breast cancer cases and unaffected controls, as described in Dorling et al.[1] and eTable 1. The analyses presented here are based on data from the subset of cases from population or hospital-based studies and controls that were sampled independently of family history (38 contributing studies). Only women aged between 18 and 79 years with no missing information on age were included.

Studies sampled controls from among women in the same population such that the age distribution was similar to that of the cases, without individual matching. Analyses were presented in terms of odds ratios (ORs). In the computation of cumulative risks were assumed to approximate incidence rate ratios: this is an approximation because density-based sampling was not used; however, the difference is slight because study recruitment was over a short period of time and the probability of a potential control becoming a case was small (the rare disease assumption).

Ethnicity was defined genetically using principal components analysis from the array genotype data where this was available, otherwise by self-report. For Malaysia and Singapore, we excluded admixed individuals, defined as not reaching a 50% threshold for a single ancestry (Chinese, Malay or Indian) based on genotyping. We also excluded individuals who were from a minority ancestry for that study (that is, non-east Asian individuals from the 4 Asian studies and non-European individuals from the European studies). Five countries were removed from imputation and subsequent regressions: France (missing Grade), Thailand, Belarus, and Canada (missing HER2 status), Cyprus (missing tumor size).

Tumor Pathology Data

Pathology information was based on histology and immunohistochemistry results from medical records, rescored whole slides or tumor microarrays, curated in BCAC database v12. Data obtained from individual study centers were centrally harmonized and checked according to a standard data dictionary. ER, PR and HER2 status was obtained mostly from medical records followed by immunohistochemistry performed on tumor tissue microarrays or whole-section tumor slides[2,3]. The cut-off was 10% for ER and PR for most studies; some USA based studies used a 1% cut-off. For HER2 scored by immunohistochemistry, in the majority of studies 0-2+ were categorized as negative and 3+ as positive in most studies. Some studies used FISH/CISH or SISH to confirm HER2 status. Most studies used the Bloom and Richardson (SBR) system for grading tumors. The variable 'Stage' was collated by studies individually but largely reflects TNM Staging. The European TNM staging (https://www.uicc.org/resources/tnm), which is very similar to the AJCC TNM staging, was used,. Some studies from the USA that used SEER staging, these were recoded as far as possible to TNM staging.

Patterns of missing in the pathology data are shown in eTables 5 and 6; pathology was more likely to be missing among younger women, but there was no correlation between missingness and genotype.

Laboratory Methods, Variant calling and classification

Details of library preparation and sequencing procedures are described in Dorling *et al.*[1] Library preparation was conducted using the Fluidigm Juno 192.24 system. Amplified products were combined into barcoded libraries of 768 samples, which were run on a single lane of an Illumina Hiseq4000. Samples were demultiplexed and then aligned to the reference genome (hg19) using BWA-MEM[4]. Each sample was sequenced to an average depth of 349 reads, in the target region. Depth, along with base quality, was used as part of the secondary quality control filtering. Variant calling was performed using VarDict[5]; further details of variant calling, filtering and quality control are given in Dorling *et al.*[1]

Variants were defined as PTVs if they were frameshifting insertions/deletions, stop-gain single nucleotide variants or canonical splice variants, with the exception of variants in the last exon of each gene and some canonical splice variants that may not be protein truncating. We also analyzed rare missense variants in *BRCA1*, *BRCA2* and *TP53* classified as pathogenic according to clinical guidelines. For *BRCA1* and *BRCA2* we considered variants classified as (likely) pathogenic using the ENIGMA *BRCA1/2* expert panel guidelines (https://enigmaconsortium.org/), or by clinical testing laboratory submitters to ClinVar (https://www.ncbi.nlm.nih.gov/clinvar/) which largely employ adaptations of the American College of Medical Genetics (ACMG) guidelines[5]. For *TP53*, we considered a definition of (likely) pathogenic, based on ACMG guidelines[6], augmented by variants classified as (likely) pathogenic based on a published quantitative model for

TP53 missense variant classification that utilizes a combination of bioinformatic prediction and the reported somatic:germline ratio for a given variant[1,7].

~80% of *CHEK2* PTVs were c.1100delC. *TP53* PTV and MSV carriers were considered together.

Five women carrying PTVs in both *BRCA1* and *BRCA2* and 11 women carrying PTVs in more than one 'non*BRCA1/2'* gene were excluded from these analyses. Women harbouring mutations in *BRCA1* or *BRCA2* plus a non-*BRCA* gene were included in the *BRCA1* and *BRCA2* analysis respectively, consistent with Dorling et al.[1] As numbers of such double mutations are very small compared with total numbers of *BRCA1* and *BRCA2* carriers, there was a trivial difference in the results when in sensitivity analyses these women excluded (data not shown). Specifically, 8 women carrying a *BRCA1* PTV and a PTV in a non*BRCA1/2* gene and one woman carrying a *BRCA1* PTV and TP53 MSV were included only in the *BRCA1* PTV analysis; 18 women carrying a *BRCA2* PTV and a PTV in a non*BRCA1/*2 gene and one woman carrying a *BRCA2* PTV and a *BRCA2* MSV were included only in the *BRCA2* PTV analysis. There was little difference in the results in sensitivity analyses of the association between intrinsic subtypes and *BRCA1* or *BRCA2* mutation status that excluded these women.

Imputation using MICE and an EM-algorithm

To evaluate heterogeneity of risk by intrinsic tumor subtypes, we used Multiple Imputation by Chained Equations (MICE) to impute missing pathology variables. ER, PR, HER2, grade, tumor size, lymph node involvement, country, age and the presence or absence of PTV or MSV in the BC genes were used to inform imputations. Missing data patterns and diagnostics for multiple imputation were inspected (data not shown). Intrinsic subtypes were constructed for each of 100 imputed datasets and results of multinomial regression for each imputed dataset pooled.

For some analyses, we also used a polytomous regression approach (TOP) which iteratively imputes pathology characteristics using an EM algorithm and has improved power for identifying heterogenous associations between risk loci and tumor subtypes.[8] When implementing TOP, we imputed only ER, PR, HER2 and Grade. Countries with missing information for >10 individuals for two or more tumor markers were excluded from the analyses.

MICE is the most widely used imputation method and provided the flexibility required to conduct all the analyses. The EM approach should converge to the maximum likelihood estimate, whereas the MICE approach relies on random resampling. As MICE is a well-established method with robust properties, and the results were very consistent where both methods were used, we used MICE as the standard approach.

Estimating Odds ratios for association between PTV/MSV carrier status and intrinsic subtypes

Multinomial logistic regression was used to estimate the odds ratios (ORs) associated with carrying any PTV (or pathogenic MSV) in each gene. Age interactions were evaluated by fitting an age x gene interaction term in the model. Subtype-specific age-interaction terms were meta-analyzed and Wald test p-values for the combined interaction ORs calculated.

Calculation of cumulative risk of developing BC subtypes

Cumulative risks for each subtype were calculated by combining age-specific ORs estimates with UK population incidence rates (2016) as baseline (https://www.cancerresearchuk.org/health-professional/cancer-statistics/statistics-by-cancer-type/breast-cancer/incidence-invasive), accounting for competing risk of not developing BC of a different subtype[9]. For these computations, the ORs were assumed to approximate the incidence rate ratios (i.e. the rare disease assumption). PTVs in *ATM*, *BARD1*, *BRCA1*, *BRCA2*, *CHEK2*, *PALB2*, *RAD51C* and *RAD51D* were included in the absolute risk model. Age-specific ORs were derived by assuming a linear trend in the log(OR) with age for all subtypes apart from *ATM*, *BARD1*, *RAD51C*, and *RAD51D*. For *BRCA1, BRCA2*, and *PALB2* a model assuming the same age-trend in each subtype was assumed. For *CHEK2* triple-negative disease no age interaction was assumed, while for all other subtypes the model assumed the same age-trend in each subtype. Where the same age-trend was assumed, the effect size based on a (fixed-effect) meta-analysis of these subtype-specific age-interaction estimates was used. The interaction effect size was included in multinomial logistic regression as an offset term to obtain the corresponding main effects coefficients.

© 2022 Breast Cancer Association Consortium. *JAMA Oncol.*

Age- and gene-specific subtype proportions for BOADICEA

For analyses carried out for inclusion of tumor subtypes in the BOADICEA risk prediction algorithm[10], the three subtypes currently considered: i) ER-positive ii) triple-negative, and iii) ER-negative but not triple-negative, were used. Age- and gene-specific subtype proportions for each tumor subtype in BOADICEA (eTable 14) were calculated by first estimating ORs for PTV carriers and the respective age-interactions for each subtype as described above. These estimates are relative to non-carriers of deleterious variants of any of the genes. Therefore, the corresponding relevant baseline subtype proportions were the proportions in non-carriers. For this, we used the non-carrier proportions in European cases in the BRIDGES analysis, to allow for possible differences in subtype proportions by ethnicity (the OR estimates were, however, from the whole dataset as there is no evidence for differences in effect size by population).

Subtype proportions were first computed in 5-year intervals, and then smoothed using Lowess, with a bandwidth of 0.2, for ER-positive, triple-negative and ER-negative non- triple-negative separately. These estimates were then further smoothed to annual proportions by assuming a linear change in proportion between the midpoint of each interval.

The proportions in each subtype were finally derived using the formula:

$$P_{sg}(t) = \frac{\lambda_{sg}(t)}{\sum_{s'} \lambda_{s'g}(t)}$$

$$= \frac{\lambda_{s0}(t) r_{sg}(t)}{\sum_{s'} \lambda_{s'0}(t)\, r_{s'g}(t)}$$

$$P_{sg}(t) = \frac{P_{s0}(t) r_{sg}(t)}{\sum_{s'} P_{s'0}(t)\, r_{s'g}(t)}$$

Where $P_{sg}(t)$ is the proportion of cases at time t in subtype s, $\lambda_{sg}(t)$ is the incidence of subtype s for gene g at time t and $r_{sg}(t)$ is the relative risk (OR) at time t, relative to gene category 0 (i.e. non-carriers).

© 2022 Breast Cancer Association Consortium. *JAMA Oncol.*

eTable1. Description of studies included in the analyses

Study	Abbreviation	Country	Study design	Case definition	Cases	Control definition	Controls	Selected familial cases	Design category	References
Amsterdam Breast Cancer Study	ABCS	Netherlands	Hospital-based consecutive cases; population-based controls (for iCOGS/OncoArray/BRIDGES from blood bank).	iCOGS/OncoArray/BRIDGES:<br>Breast cancer patients diagnosed before age 50 in 1995-2011 at the Netherlands Cancer Institute - Antoni van Leeuwenhoek hospital (NKI-AVL).	992	iCOGS/OncoArray/BRIDGES: Population-based cohort of women recruited through the Sanquin blood bank, all ages.	1408	No	Mixed	[11,12]
Asia Cancer Program	ACP	Thailand	Hospital-based case-control study	Cases recruited 1999-2000 and 2008-present at The National Cancer Institute (Central region), The Prince Songkla University Research Centre (South region), The HRH Princess Maha Chakri Sirindhorn Medical Centre (MSMC)-Srinakarinviroj University (Eastern region), Khon-Kaen University Cancer Centre (North-eastern region).<br>1. Women who underwent biopsy and have been pathologically diagnosed as having breast cancer.<br>2. Aged less than 71 years of age.	601	Controls recruited 1999-2000 and 2008-present at The National Cancer Institute (Central region), The Prince Songkla University Research Centre (South region), The HRH Princess Maha Chakri Sirindhorn Medical Centre (MSMC)-Srinakarinviroj University (Eastern region), Khon-Kaen University Cancer Centre (North-eastern region).<br>1. Women aged less than 71 years of age without cancer history of any kinds<br>2. Women who attend the out-patient clinic under the minor injuries such as cuts, broken bones.<br>3. Women who are institutionalised at the hospital with diseases	557	No	Mixed	None

© 2022 Breast Cancer Association Consortium. *JAMA Oncol.*

Study	Abbreviation	Country	Study design	Case definition	Cases	Control definition	Controls	Selected familial cases	Design category	References
						not related to cancer or metabolic syndromes such as diabetes, heart diseases or conditions related to gynaecology and are well enough to give information to researchers.				
Bavarian Breast Cancer Cases and Controls	BBCC	Germany	Hospital-based cases; population based controls	Consecutive, unselected cases with invasive breast cancer recruited at the University Breast Centre, Franconia in Northern Bavaria during 1999-2013.	216	Healthy women with no diagnosis of cancer aged 55 or older. Invited by a newspaper advertisement in Northern Bavaria and recruited during 1999-2013.	157	No	Mixed	[13,14]
Breast Cancer in Galway Genetic Study	BIGGS	Ireland	Hospital-based cases; population based controls	Unselected cases recruited from West of Ireland since 2001. Cases were recruited from University College Hospital Galway and surrounding hospitals	344	Women > 60 years with no personal history of any cancer and no family History of breast or ovarian cancer were identified from retirement groups in the West of Ireland (same catchment area as cases) during the period 2001-2008.	21	No	Mixed	[15-17]
Breast Oncology Galicia Network	BREOGAN	Spain	Population-based case-control	A population-based study conducted since 1997 in two cities in Galicia, Spain (Vigo and Santiago) covering approximately 700,000 inhabitants. The study currently includes over 1600 incident breast cancer cases diagnosed from 1997-2014 in two	521	Controls were frequency-matched to cases according to 5-year age group, inclusion in the universal Galician Public Health Service (SERGAS) registry database, and place of residence. They were healthy, unrelated female individuals from	388	No	Population-based	[17-21]

© 2022 Breast Cancer Association Consortium. *JAMA Oncol.*

Study	Abbreviation	Country	Study design	Case definition	Cases	Control definition	Controls	Selected familial cases	Design category	References
				Galician hospitals with blood, tumor tissue and risk factor questionnaire.		the same base population as cases randomly selected from SERGAS´ primary healthcare centers in the health areas of Santiago and Vigo. Recruitment began in 1997.				
Breast Cancer Study of the University of Heidelberg	BSUCH	Germany	Hospital-based cases;healthy blood donator controls	Cases diagnosed with breast cancer/breast cancer metastasis in 2008-2011 at the University Women`s Clinic Heidelberg.	226	Healthy, unrelated, ethnically matched female blood donors recruited in 2007, 2009 & 2012 by German Red Cross Blood Service of Baden-Württemberg-Hessen, Institute of Transfusion Medicine & Immunology, Mannheim.	549	No	Mixed	[22]
Crete Cancer Genetics Program	CCGP	Greece	Hospital-based case-control study	Incident breast cancer cases treated between 2004 and 2013 at the University Hospital of Heraklion on Crete; all enrolled within 6 months of diagnosis.	428	Healthy, unrelated, ethnically matched female blood donors recruited in 2014 by the laboratory of Hemostasis at the General Hospital of Heraklion "Venizelio".	217	No	Mixed	Unpublished
CECILE Breast Cancer Study	CECILE	France	Population-based case-control study	All incident cases of breast cancer diagnosed in 2005-2007 among women <75 years of age and residing in Ille-et-Vilaine or Côte d'Or. Cases were recruited from the main cancer treatment center (Centre Eugène-Marquis in Rennes and Centre	832	General population control women residing in the same geographic areas frequency-matched to the cases by 5-year age groups. Controls were recruited in 2005-2007 by phone using a random digit dialing procedure and predefined numbers by	943	No	Population-based	[23]

© 2022 Breast Cancer Association Consortium. *JAMA Oncol.*

Study	Abbreviation	Country	Study design	Case definition	Cases	Control definition	Controls	Selected familial cases	Design category	References
				Georges-François-Leclerc in Dijon) and from private or public hospitals in each area.		socioeconomic status to control for possible selection bias.				
Copenhagen General Population Study	CGPS	Denmark	Population-based case-control study	Consecutive, incident cases from 1 hospital with centralized care for a population of 400,000 women from 2001 to the present.	2988	Community controls residing in the same region as cases and with no history of breast cancer were identified from the Copenhagen General Population Study recruited 2003-2007. All controls were known to still be breast cancer-free at the end of 2007.	4920	No	Mixed	[24]
Spanish National Cancer Centre Breast Cancer Study	CNIO-BCS	Spain	Case-control study	Two groups of cases:1) 574 consecutive breast cancer patients, unselected for family history, from 3 public hospitals, 2 in Madrid and one in Oviedo, from 2000 to 2005. 2) 291 cases with at least one first degree relative also affected with breast cancer, recruited through the CNIO family cancer clinic in Madrid from 2000 to 2004.	402	Women attending the Menopause Research Centre between 2000 and 2004 and female members of the College of Lawyers attending a free, targeted medical check-up in 2005, all free of breast cancer and all in Madrid	557	Subset (N=291)	Mixed	[25]

© 2022 Breast Cancer Association Consortium. *JAMA Oncol.*

Study	Abbreviation	Country	Study design	Case definition	Cases	Control definition	Controls	Selected familial cases	Design category	References
Colombian Breast Cancer Case-Control Study	COLBCCC	Colombia	Case-control study	1,022 unselected women diagnosed with breast cancer after January 1, 2004; enrolled between 2007 and 2012.	370	1,023 healthy women attending the country-wide National Pap-Smear Screening Program in Colombia; enrolled between 2007 and 2012. Controls were matched to cases by +/- 2 years. Controls were women participating in the Colombian National Pap-Smear Screening Program (participation rate in 2005 was 77%)	614	No	Mixed	Unpublished
German Consortium for Hereditary Breast & Ovarian Cancer	GC-HBOC	Germany	Clinic-based case study and prospective cohort study	Women diagnosed with breast cancer in one of the GC-HBOC centers (Cologne, Munich, Kiel, Heidelberg, Düsseldorf, Ulm, Würzburg, Münster and Hannover). Recruitment period 1996-present.	0	Healthy, unrelated, ethnically and age-matched female control individuals (LIFE study, Leipzig, Germany).	1561	Yes	Mixed	[26-29]
Gene Environment Interaction and Breast Cancer in Germany	GENICA	Germany	Population-based case-control study	Incident breast cancer cases enrolled between 2000 and 2004 from the Greater Bonn area (by of the hospitals within the study region); all enrolled within 6 months of diagnosis.	806	Selected from population registries from 31 communities in the greater Bonn area; matched to cases in 5-year age classes between 2001 and 2004.	891	No	Population-based	[30,31]

© 2022 Breast Cancer Association Consortium. *JAMA Oncol.*

Study	Abbreviation	Country	Study design	Case definition	Cases	Control definition	Controls	Selected familial cases	Design category	References
Generation Scotland	GENSCOT	Scotland	Prospective family-based cohort study; nested case-control	Incident and prevalent cases of histologically-confirmed breast cancer at the time of latest updated cancer registry linkage (currently 2013). Recruitment though the General Practitioners in the areas of Glasgow, Tayside, Ayrshire, Arran and Northeast Scotland.	384	Two groups of controls: (1) 2:1 unrelated individuals matched to cases on age in five-years at baseline and recruitment centre; (2) first-degree female relatives with no breast cancer diagnosis at the time of selection.	746	No	Prospective cohort	[32]
Genetic Epidemiology Study of Breast Cancer by Age 50	GESBC	Germany	Population-based study of women <50 years	All incident cases diagnosed <50 years of age in 1992-5 in two regions: Rhein-Neckar-Odenwald and Freiburg, by surveying the 38 clinics serving these regions	498	Selected from random lists of residents of the study regions supplied by population registries; two controls were selected for each case, matched by age and study region. Recruitment was carried out 1992-1998.	982	No	Population-based	[33]
Hannover Breast Cancer Study	HABCS	Germany	Hospital-based case-control study	Cases who received radiotherapy for breast cancer at Hannover Medical School between 1996-2003 (HaBCS I), or were diagnosed with breast cancer at a certified Breast Cancer Clinics in the Hannover region between 2012-2016 (HaBCS II), unselected for age or family history.	819	Anonymous female blood bank donors at Hannover Medical School, collected from 8/2005-12/2005, with known age and ethnic background.	833	No	Mixed	[34]

© 2022 Breast Cancer Association Consortium. *JAMA Oncol.*

Study	Abbreviation	Country	Study design	Case definition	Cases	Control definition	Controls	Selected familial cases	Design category	References
Helsinki Breast Cancer Study	HEBCS	Finland	Hospital-based case-control study, plus additional familial cases	(1) Consecutive cases (883) from the Department of Oncology, Helsinki University Central Hospital 1997-8 and 2000, (2) Consecutive cases (986) from the Department of Surgery, Helsinki University Central Hospital 2001 – 2004, (3) Familial breast cancer patients (536) from the Helsinki University Central Hospital, Departments of Oncology and Clinical Genetics (1995-)	1240	Healthy females from the same geographical region in Southern Finland in 2003.	1090	Subset (N=609)	Mixed	35-37
Hannover-Minsk Breast Cancer Study	HMBCS	Belarus	Hospital-based cases; population based controls	Ascertainment at the Byelorussian Institute for Oncology and Medical Radiology Aleksandrov N.N. in Minsk or at one of 5 regional oncology centers in Gomel, Mogilev, Grodno, Brest or Vitebsk through the years 2002-2008.	332	Controls from the same population aged 18-72 years. Healthy (without personally history of cancer) female probunds recruited from the same geographical regions as cases during the years 2002-2008. About 75% of controls were women invited for general medical examination at five regional gynecology clinics (in Gomel, Mogilev, Grodno, Brest or Vitebsk) and cancer-free volunteers ascertained at the Institute for Inherited Diseases in Minsk; 20%	267	No	Mixed	38

© 2022 Breast Cancer Association Consortium. *JAMA Oncol.*

Study	Abbreviation	Country	Study design	Case definition	Cases	Control definition	Controls	Selected familial cases	Design category	References
						were cancer-free female blood bank donors recruited at Republic Blood Bank, Minsk, Belarus; finally 5% of controls were healthy cancer-free relatives of some breast cancer patients.				
Hannover-Ufa Breast Cancer Study	HUBCS	Russia	Hospital-based cases; population based controls	Consecutive Russian breast cancer patients aged 24-86 years ascertained at one of the two participating oncological centers in Bashkorstostan and Siberia through the years 2000-2008.	224	Population controls aged 18-84 years recruited from a population study of different populations of Russia. Healthy volunteers (without any malignancy) were selected from the same geographical regions during the years 2002-2008.	188	No	Mixed	[38]
Karolinska Breast Cancer Study	KARBAC	Sweden	Population and hospital-based cases; geographically matched controls	1. Familial cases from Department of Clinical Genetics, Karolinska University Hospital, Stockholm. 2. Consecutive cases from Department of Oncology, Huddinge & Söder Hospital, Stockholm 1998-2000	287	Blood donors of mixed gender from same geographical region. Excess material was received from all blood donors over a 3 month period in 2004 (approximately 3000) and DNA was extracted from a random sample of 1500	0	Subset (N=568)	Mixed	[39,40]

© 2022 Breast Cancer Association Consortium. *JAMA Oncol.*

Study	Abbreviation	Country	Study design	Case definition	Cases	Control definition	Controls	Selected familial cases	Design category	References
Karolinska Mammography Project for Risk Prediction of Breast Cancer - Cohort Study	KARMA	Sweden	Cohort study	Inclusion of 70,877 women Oct 2010 - March 2013. 3000 women had BC at cohort entry. In all, 800 women have been diagnosed with breast cancer since study entry (Oct 2015). Approximately 250 women are diagnosed with BC annually	2953	Non - BC cases in the Karma Cohort	5626	no	Prospective cohort	Submitted
Kuopio Breast Cancer Project	KBCP	Finland	Population-based prospective clinical cohort	1. Women seen at Kuopio University Hospital between 1990 and 1995 because of breast lump, mammographic abnormality, or other breast symptom who were found to have breast cancer. 2. Consecutive malignant breast cancer cases diagnosed at KUH from 2011 onwards.	476	Age and long-term area-of-residence matched controls selected from the National Population Register and interviewed in parallel with the cases	70	No	Population-based	[41,42]
Kathleen Cuningham Foundation Consortium for research into Familial Breast Cancer/Australian Ovarian Cancer Study	kConFab/AOCS	Australia and New Zealand	Clinic-based recruitment of familial breast cancer patients (cases); population-based case-control study of ovarian cancer (controls only)	Cases were from multiple-case breast and breast-ovarian families recruited though family cancer clinics from across Australia and New Zealand from 1998 to the present. Cases were selected for inclusion in BCAC studies if (i) family was negative for mutations in	0	Female controls were ascertained by the Australian Ovarian Cancer Study identified from the electoral rolls from all over Australia from 2002-2006.	7	Yes	Mixed	[43,44]

© 2022 Breast Cancer Association Consortium. *JAMA Oncol.*

Study	Abbreviation	Country	Study design	Case definition	Cases	Control definition	Controls	Selected familial cases	Design category	References
				BRCA1 and BRCA2 (ii) case was the index for the family, defined as youngest breast cancer affected family member.						
Mammary Carcinoma Risk Factor Investigation	MARIE	Germany	Population-based case-control study	Incident cases diagnosed from 2001-2005 in the study region Hamburg in Northern Germany, and from 2002-2005 in the study region Rhein-Neckar-Karlsruhe in Southern Germany.	2085	2 controls per case were randomly drawn from population registries and frequency matched by birth year and study region to the case. Controls were recruited from 2002 to 2006.	1768	No	Population-based	[45]
Cyprus Breast Cancer Case Control Study	MASTOS	Cyprus	Population-based case-control study	Women between 40-70 years of age who had a histologically confirmed diagnosis of primary breast cancer between January 1999 and December of 2005. The majority of cases were ascertained from the Bank of Cyprus Oncology Centre, which operates as a referral centre and offers treatment and follow-up for up to 90% of all breast cancer cases diagnosed in Cyprus.	656	Cypriot women from the general population, who were invited to participate in the National programme for breast cancer screening with the use of mammography and received a negative result. Volunteers were enrolled in the study during the same calendar period as the cases, from the 5-district mammography screening centers that operate in Cyprus.	1091	No	Population-based	[46]

© 2022 Breast Cancer Association Consortium. *JAMA Oncol.*

Study	Abbreviation	Country	Study design	Case definition	Cases	Control definition	Controls	Selected familial cases	Design category	References
				The rest of the patients, were recruited at the Oncology Departments of the Nicosia, Limassol, Larnaca and Paphos district hospitals.						
Melbourne Collaborative Cohort Study	MCCS	Australia	Prospective cohort study: nested case-control study	Incident cases diagnosed between baseline (1990-1994) and last follow-up (2012) among the 24469 women participating in the cohort.	793	For each case a control was randomly selected from women from the cohort who did not develop breast cancer before the age at diagnosis of the case and matched the case on year of birth and country of birth.	971	No	Prospective cohort	[47]
Malaysian Breast Cancer Genetic Study	MYBRCA	Malaysia	Hospital-based case-control study	Breast cancer cases identified at the Breast Cancer Clinic in University Malaya Medical Centre Jan 2003-July 2014 and Subang Jaya Medical Centre Sep 2012-Sept 2014; cases are a mixture of prevalent and incident cases. Includes hospital-based and familial series.	823	Controls are cancer-free individuals (37-74 years) selected from women attending mammographic screening at the same hospitals.	1090	Yes (subset)	Mixed	[48,49]

© 2022 Breast Cancer Association Consortium. *JAMA Oncol.*

Study	Abbreviation	Country	Study design	Case definition	Cases	Control definition	Controls	Selected familial cases	Design category	References
Norwegian Breast Cancer Study	NBCS	Norway	Hospital-based case-control study	Incidence cases from three different hospitals: 1) Cases (114) mean age 64 (28-92) at Ullevål Univ. Hospital 1990-94, 2) cases (182) mean age 59 (26-75) referred to Norwegian Radium Hospital 1975-1986, 3) cases (124), mean age 56 (29-82) with stage I or II disease, in the Oslo micro-metastases study at Norwegian Radium Hospital between 1995-1998, 4) Breast cancer cases referred to the Norwegian hospitals Akershus University Hospital in Lørenskog, Ullevaal university hospital in Oslo and Rikshospitalet-Radiumhospitalet in Oslo from 2007-2010. Mean age is 63 years. Consecutive series. 5) Breast cancer cases referred to the Norwegian Radium Hospital hospitalet 2010-2013. Neoadjuvantly treated with Avastin (Bevacizumab). 6) Consecutive series of Breast cancer incidents referred to Akershus	436	Control subjects were healthy women, age 55-71, residing in Tromsø (440), and Bergen (109) attending the Norwegian Breast Cancer Screening Program. Healthy tissue from mammoplastic reduction surgery at a private clinic in Oslo.	597	No	Mixed	[50-53]

© 2022 Breast Cancer Association Consortium. *JAMA Oncol.*

Study	Abbreviation	Country	Study design	Case definition	Cases	Control definition	Controls	Selected familial cases	Design category	References
				university hospital 2004-2014.						
Ontario Familial Breast Cancer Registry	OFBCR	Canada	Population-based familial case-control study	Cases diagnosed between 1 Jan 1996-31 Dec 1998 were identified from the Ontario Cancer Registry which registers >97% of all cases residing in the province at the time of diagnosis. All women with invasive breast cancer aged 20–54 years who met the	108	Unrelated, unaffected population controls were recruited by the Ontario Familial Breast and Colon Cancer Registries by calling randomly selected residential telephone numbers throughout the same geographical region. Eligible controls were	415	Subset (N=628)	Mixed	[54]

© 2022 Breast Cancer Association Consortium. *JAMA Oncol.*

Study	Abbreviation	Country	Study design	Case definition	Cases	Control definition	Controls	Selected familial cases	Design category	References
				OFBCR definition for high genetic risk (family history of specific cancers particularly breast and ovarian, early onset disease, Ashkenazi ethnicity or a diagnosis of multiple breast cancer) were asked to participate by completing risk factor questionnaires and providing a blood sample. A 25% random sample of individuals in this age category who did not meet the OFBCR definition, 35% of those aged 55–69 at high risk and 8.75% aged 55–69 at low risk were also asked to participate. Individuals diagnosed in 2001 and 2002 were also included if they met high-risk criteria.		women with no history of breast cancer and were frequency-matched by 5-year age group to the expected age distribution of cases.				
NCI Polish Breast Cancer Study	PBCS	Poland	Population-based case-control study	Incident cases from 2000-2003 identified through a rapid identification system in participating hospitals covering ~ 90% of all eligible cases, and cancer registries in Warsaw and Łódź covering 100% of all eligible cases.	1564	Randomly selected from population lists of all residents of Poland, stratified and frequency matched to cases by case city and age in 5 year categories. Recruited 2000-2003.	1849	No	Population-based	[55]

© 2022 Breast Cancer Association Consortium. *JAMA Oncol.*

Study	Abbreviation	Country	Study design	Case definition	Cases	Control definition	Controls	Selected familial cases	Design category	References
The Prostate,Lung,Colorectal and Ovarian (PLCO) Cancer Screening Trial	PLCO	USA	Prospective cohort study: nested case-control	Incident cases arising in the sub-cohort of 78,232 women who gave a blood specimen in 1993-2001 are included if they were diagnosed with breast cancer. Recruitment via multiple screening centers across the US.	1530	Controls were women in this sub-cohort who were not diagnosed with breast cancer. Controls were matched to cases on age at randomization (4 categories) and fiscal year of randomization (2 categories).	2221	No	Prospective cohort	[56]
Predicting the Risk Of Cancer At Screening Study	PROCAS	UK	Population based study	Women diagnosed with breast cancer since joining the study of women attending the Breast Screening Programme (NHSBSP) in Greater Manchester. Recruitment period Oct 2009-May 2014.	297	Women attending routine NHS breast screening in Greater Manchester without a breast cancer diagnosis. Recruited during the same period as for the cases.	1434	No	Population-based	[57]
Singapore and Sweden Breast Cancer Study	SASBAC	Sweden	Population-based case-control study	Incident cases from October 1993 to March 1995 identified via the 6 regional cancer registries in Sweden, to which reporting is mandatory.	1110	Controls were randomly selected from the total population registry in 5-year age groups to match the expected age-frequency distribution among cases. Patients and controls were recruited from Oct 1993 through April 1995.	1321	No	Population-based	[58]
Study of Epidemiology and Risk factors in Cancer Heredity	SEARCH	UK	Population-based case-control study	2 groups of cases identified through East Anglian Cancer Registry; 1) prevalent cases diagnosed 1991-1996 under 55 years of age at diagnosis, recruited 1996-2002; 2) incident	12387	Two groups of controls: (1) selected from the EPIC-Norfolk cohort study of 25,000 individuals age 45-74 recruited between 1992 and1994, based in the same geographic region	6414	No	Mixed	[59]

© 2022 Breast Cancer Association Consortium. *JAMA Oncol.*

Study	Abbreviation	Country	Study design	Case definition	Case s	Control definition	Control s	Selecte d familial cases	Design category	Reference s
				cases diagnosed since 1996 under 70 years of age at diagnosis, recruited 1996-present.		as cases; (2) selected from GP practices from March 2003 to present, frequency matched to cases by age and geographic region				
Singapore Breast Cancer Cohort	SGBCC	Singapore	Hospital-based breast cancer cohort and population-based controls	Living breast cancer patients diagnosed with primary in situ or invasive breast cancer at 7 restructured hospitals in Singapore between 1980-2016. Cases are a mixture of prevalent and incident cases.	3224	All community-dwelling individuals who are Singaporeans or Singaporean Permanent Residents, 21 years and older. Participants were recruited between 2006 and 2010 through word-of-mouth and personal recommendations. In some cases, recruiters also sought participants through "cold-calling" or through door-to-door invitations.<br>Exclusion criteria were a medical history of cancer, acute myocardial infarction or stroke, or major psychiatric morbidity including schizophrenia, psychotic depression, and advanced Alzheimer's Disease.	4165	No	Hospital-based	No refs.

© 2022 Breast Cancer Association Consortium. *JAMA Oncol.*

Study	Abbreviation	Country	Study design	Case definition	Cases	Control definition	Controls	Selected familial cases	Design category	References
Städtisches Klinikum Karlsruhe Deutsches Krebsforschungszentrum Study	SKKDKFZS	Germany	Hospital-based breast cancer cohort	Women diagnosed with primary *in situ* or invasive breast cancer at the Städtisches Klinikum Karlsruhe from March 1993 to July 2005.	859	No controls.	0	No	Patient cohort	[60]
IHCC-Szczecin Breast Cancer Study	SZBCS	Poland	Hospital-based case-control study	Prospectively ascertained cases of invasive breast cancer patients diagnosed at the Regional Oncology Hospital (Szczecin) in the years 2002, 2003, 2006 and 2007 or the University Hospital from 2002 to 2007 in Szczecin, West-Pomerania, Poland. Patients with pure intraductal or intralobular cancer were excluded (DCIS or LCIS) but patients with DCIS with micro-invasion were included.	297	Unaffected, matched to cases for year of birth, sex and region; from families with negative cancer family history; controls were part of a population-based study of the 1.3 million inhabitants of West Pomerania performed in 2003 and 2004 designed to identify familial aggregations of cancer by our centre	189	No	Mixed	[61-64]

© 2022 Breast Cancer Association Consortium. *JAMA Oncol.*

Study	Abbreviation	Country	Study design	Case definition	Cases	Control definition	Controls	Selected familial cases	Design category	References
Utah Breast Cancer Study	UBCS	USA	Mixed. (1) Pedigrees including multiple sampled breast cancer cases within 2 generations, also may include sampled, unaffected relatives; (2) hospital-based cases (from Huntsman Cancer Institute [HCI] or Intermountain Healthcare [IH]), and breast reduction controls; and (3) Population-based cases (from the Utah Cancer Registry [UCR]) and controls (from the Utah Drivers License Registry [UDLR])	Cases recruited from late 1970s to present (on-going). Ascertainment from: (1) UCR-confirmed breast cancer cases in high-risk pedigrees; (2) invasive breast cancer cases treated or surgery performed at HCI or IH clinics; (3) prevalent, population-based UCR-confirmed breast cancer cases.	572	Controls also recruited from late 1970s to present (on-going) from: (1) relatives in high-risk pedigrees; (2) hospital-based cancer-free women undergoing breast reductions; (3) Population-based controls selected from the UDLR to frequency match cases by sex and birth cohort.	270	Some	Mixed	[65,66]

© 2022 Breast Cancer Association Consortium. *JAMA Oncol.*

eTable 2: Immunohistochemistry and tumour grade - based surrogates for five intrinsic breast cancer subtypes

Intrinsic subtype	IHC surrogate	Abbreviation
Luminal A-like	ER+ and/or PR+ HER2- Grades 1 or 2	HR+HER2-lowgrade[a]
Luminal B-HER2-positive like	HER-2 positive like: ER+ and/or PR+ HER2+	HR+HER2+
Luminal B-HER2-negative like	ER+ and/or PR+ HER2- Grade 3	HR+HER2-highgrade
HER2 enriched	ER- PR- HER2+	HR-HER2+
TN	ER- PR- HER2-	TN

IHC, immunohistochemistry; HR, Hormone receptor; ER, estrogen receptor; PR, progesterone receptor; HER2, human epidermal growth factor receptor 2; TN, triple negative; +, positive; -, negative

[a] 'lowgrade' includes low (Grade 1) and intermediate (Grade 2) grade tumors

© 2022 Breast Cancer Association Consortium. *JAMA Oncol.*

eTable 3: Numbers of cases and controls, and age distributions, by country of origin

	Controls					Cases				
		Age at Interview (years)					Age at Diagnosis (years)			
Country	N	Mean	Sd	Median	IQR	N	Mean	Sd	Median	IQR
Australia	978	62.0	8.8	63	12	793	62.2	9.2	63	13
Belarus[a]	267	46.4	13.1	47	21	332	47.4	12.3	46	17
Canada[a]	415	55.0	12.0	55	18	108	57.8	8.7	61	14
Colombia	614	49.7	10.9	50	14	370	48.7	11.9	48	15
Cyprus[a]	1091	55.7	7.0	56	10	656	51.1	9.0	51	13
Denmark	4920	55.5	12.1	55	18	2988	59.5	11.0	60	17
Finland	1160	41.5	13.3	42	23	1716	56.6	11.1	56	16
France[a]	943	54.6	11.0	55	16	832	54.2	10.8	55	16
Germany	6741	54.6	13.3	57	16	5509	58.3	10.6	60	15
Greece	217	57.6	15.1	62	19	428	55.2	12.0	55	19
Ireland	21	64.6	7.3	64	10	344	51.2	10.0	50	12
Malaysia	1090	56.0	8.5	56	13	823	52.2	10.6	52	15
Netherlands	1408	47.1	12.3	48	18	992	42.1	6.0	44	8
Norway	597	61.4	4.5	60	7	436	58.3	10.4	59	15
Poland	2038	55.7	9.9	55	16	1861	56.1	10.0	55	15
Russia	188	45.3	13.9	45	16	224	52.3	9.8	52	13
Singapore	4165	50.1	10.2	50	14	3224	53.2	10.1	53	14
Spain	945	51.8	11.8	53	17	923	55.6	11.0	56	16
Sweden	6947	60.8	8.8	62	13	4350	57.5	9.8	58	14
Thailand[a]	557	41.7	10.5	43	15	601	48.2	9.1	48	13
UK	8594	54.9	11.3	56	13	13068	54.6	9.0	55	13
USA	2491	61.3	7.7	61	9	2102	64.2	9.6	66	11
TOTAL	46387	55.1	11.9	56	16	42680	55.8	10.6	56	16

N, number of cases or controls; sd, standard deviation; IQR, interquartile range
[a] Five countries: Belarus, Canada, Cyprus, France and Thailand were excluded from imputation due to limited numbers for some pathology variables; A total of 43114 controls and 40151 cases were included

© 2022 Breast Cancer Association Consortium. *JAMA Oncol.*

eTable 4: Numbers of variant carriers by breast cancer susceptibility gene

Gene	Controls			Cases			Controls[a]	Cases[a]
	All ages	≤ 50 years	> 50 years	All ages	≤ 50 years	> 50 years	All ages	All ages
Non-carriers	45,633	14,484	31,149	40,108	12,538	27,570	42,399	37,728
Carriers	754	263	491	2572	1232	1340	715	2423
ATM PTV	136	52	84	263	90	173	130	250
BARD1 PTV	27	11	16	56	22	34	24	52
BRCA1 PTV	56	24	32	465	324	141	51	431
BRCA2 PTV	126	46	80	678	354	324	115	625
CHEK2 PTV	275	89	186	628	229	399	268	610
PALB2 PTV	52	18	34	245	94	151	49	233
RAD51C PTV	26	8	18	43	11	32	26	42
RAD51D PTV	25	8	17	46	15	31	23	45
BRCA1 MSV	4	0	4	58	39	19	4	56
BRCA2 MSV	7	3	4	39	20	19	7	37
TP53 PTV MSV	20	4	16	51	34	17	18	42
Total	46,387	14,747	31,640	42,680	13,770	28,910	43,114	40,151

PTV, protein truncating variants; MSV, missense variants. For TP53, too few PTVs (7) were available for separate analysis and these were combined with the deleterious missense variants; PTV and MSV occurred together in one individual

[a] Numbers of cases and controls included in imputation and analyses of intrinsic subtypes (five countries: Belarus, Canada, Cyprus, France and Thailand were excluded)

© 2022 Breast Cancer Association Consortium. *JAMA Oncol.*

eTable 5: Cross tabulation of ER, PR, HER2 and Grade data

		ER-status						PR-status						HER2-status						Grade							
	Marker status	Negative		Positive		Unknown		Negative		Positive		Unknown		Negative		Positive		Unknown		Grade 1		Grade 2		Grade 3		Unknown	
**ER-status**		N	%	N	%	N	%	N	%	N	%	N	%	N	%	N	%	N	%	N	%	N	%	N	%	N	%
	Negative	6607	100%	-	-	-	-	5066	56%	776	4%	765	6%	3078	17%	1584	37%	1945	11%	254	4%	1619	10%	4003	39%	731	10%
	Positive	-		26480		-		3916	43%	17380	96%	5184	40%	15173	83%	2698	63%	8609	49%	5576	85%	12958	80%	5246	51%	2700	38%
	Unknown	-		-		7064		28	0%	34	0%	7002	54%	132	1%	26	1%	6906	40%	737	11%	1588	10%	1039	10%	3700	52%
**PR-status**																											
	Negative	5066	77%	3916	15%	28	0%	9010	100%	-	-	-	-	4945	27%	2099	49%	1966	11%	870	13%	3015	19%	4408	43%	717	10%
	Positive	776	12%	17380	66%	34	0%	-	-	18190	100%	-	-	11881	65%	1904	44%	4405	25%	4051	62%	9166	57%	3634	35%	1339	19%
	Unknown	765	12%	5184	20%	7002	99%	-	-	-	-	12951	100%	1557	8%	305	7%	11089	64%	1646	25%	3984	25%	2246	22%	5075	71%
**HER2-status**																											
	Negative	3078	47%	15173	57%	132	2%	4945	55%	11881	65%	1557	12%	18383	100%	-	-	-	-	3874	59%	8824	55%	4775	46%	910	13%
	Positive	1584	24%	2698	10%	26	0%	2099	23%	1904	10%	305	2%	-	-	4308	100%	-	-	285	4%	1634	10%	2111	21%	278	4%
	Unknown	1945	29%	8609	33%	6906	98%	1966	22%	4405	24%	11089	86%	-	-	-	-	17460	100%	2408	37%	5707	35%	3402	33%	5943	83%
**Grade**																											
	Grade 1	254	4%	5576	21%	737	10%	870	10%	4051	22%	1646	13%	3874	21%	285	7%	2408	14%	6567	100%	-	-	-	-	-	-
	Grade 2	1619	25%	12958	49%	1588	22%	3015	33%	9166	50%	3984	31%	8824	48%	1634	38%	5707	33%	-	-	16165	100%	-	-	-	-
	Grade 3	4003	61%	5246	20%	1039	15%	4408	49%	3634	20%	2246	17%	4775	26%	2111	49%	3402	19%	-	-	-	-	10288	100%	-	-
	Unknown	731	11%	2700	10%	3700	52%	717	8%	1339	7%	5075	39%	910	5%	278	6%	5943	34%	-	-	-	-	-	-	7131	100%

PTV, protein truncating variants; MSV, missense variants; ER, estrogen receptor; PR, progesterone receptor; HER2, human epidermal growth factor receptor 2; N, number of cases; % of each column.
Missing as a % of the total: ER-status, 17.59%; PR-status, 32.36%; HER2-status, 43.49%; Grade, 17.76%

© 2022 Breast Cancer Association Consortium. *JAMA Oncol.*

eTable 6: Distribution of intrinsic tumor subtypes in women of all ages and in different age groups, by breast cancer susceptibility gene

	HR+HER2-lowgrade		**HR+HER2+**		**HR+HER2-highgrade**		**HR-HER2+**		**TN**		
	N	**Proportion**	***N***	**Proportion**	***N***	**Proportion**	***N***	**Proportion**	***N***	**Proportion**	**Total**
All ages											
Non-carriers	2171576	0.576	459426	0.122	522590	0.139	221843	0.059	397365	0.105	3772800
BRCA1 PTV	6460	0.150	1258	0.029	6800	0.158	2943	0.068	25639	0.595	43100
BRCA2 PTV	27046	0.433	6678	0.107	15576	0.249	2196	0.035	11004	0.176	62500
ATM PTV	12717	0.509	2355	0.094	7886	0.315	641	0.026	1401	0.056	25000
CHEK2 PTV	36230	0.594	8919	0.146	9720	0.159	2944	0.048	3187	0.052	61000
PALB2 PTV	8739	0.375	3130	0.134	5909	0.254	1521	0.065	4001	0.172	23300
RAD51C PTV	1603	0.382	47	0.011	716	0.170	270	0.064	1564	0.372	4200
RAD51D PTV	1500	0.333	141	0.031	1458	0.324	40	0.009	1361	0.302	4500
BARD1 PTV	2269	0.436	88	0.017	344	0.066	394	0.076	2105	0.405	5200
BRCA1 MSV	1138	0.203	197	0.035	894	0.160	373	0.067	2998	0.535	5600
BRCA2 MSV	1327	0.359	320	0.086	1397	0.378	111	0.030	545	0.147	3700
TP53 PTV MSV	1217	0.290	1410	0.336	742	0.177	540	0.129	291	0.069	4200
Total	2271822		483969		574032		233816		451461		4015100
**≤40 years**											
Non-carriers	100989	0.393	44491	0.173	44873	0.175	25189	0.098	41158	0.160	256700
BRCA1 PTV	1268	0.086	505	0.034	1846	0.126	904	0.061	10177	0.692	14700
BRCA2 PTV	4939	0.380	1968	0.151	2987	0.230	542	0.042	2564	0.197	13000
ATM PTV	623	0.389	167	0.104	698	0.436	22	0.014	90	0.056	1600
CHEK2 PTV	3304	0.501	1107	0.168	1368	0.207	526	0.080	295	0.045	6600
PALB2 PTV	733	0.333	359	0.163	595	0.270	100	0.045	413	0.188	2200
RAD51C PTV	25	0.063	4	0.010	43	0.108	13	0.033	315	0.788	400
RAD51D PTV	0	0.000	3	0.008	145	0.363	20	0.050	232	0.580	400
BARD1 PTV	202	0.404	26	0.052	24	0.048	27	0.054	221	0.442	500
BRCA1 MSV	202	0.144	43	0.031	255	0.182	6	0.004	894	0.639	1400
BRCA2 MSV	204	0.291	27	0.039	319	0.456	41	0.059	109	0.156	700
TP53 PTV MSV	251	0.157	677	0.423	76	0.048	421	0.263	175	0.109	1600
Total	112740		49377		53229		27811		56643		299800

© 2022 Breast Cancer Association Consortium. *JAMA Oncol.*

	HR+HER2-lowgrade		HR+HER2+		HR+HER2-highgrade		HR-HER2+		TN		
	N	Proportion	*N*	Proportion	*N*	Proportion	*N*	Proportion	*N*	Proportion	Total
**41-60 years**											
Non-carriers	1188674	0.567	259858	0.124	292277	0.139	129734	0.062	225757	0.108	2096300
BRCA1 PTV	4242	0.174	632	0.026	4266	0.175	1772	0.073	13488	0.553	24400
BRCA2 PTV	16129	0.441	3299	0.090	10195	0.279	990	0.027	5987	0.164	36600
ATM PTV	7909	0.531	1570	0.105	4167	0.280	380	0.026	874	0.059	14900
CHEK2 PTV	20495	0.584	5195	0.148	5870	0.167	1724	0.049	1816	0.052	35100
PALB2 PTV	5032	0.354	1869	0.132	3824	0.269	997	0.070	2478	0.175	14200
RAD51C PTV	556	0.253	27	0.012	571	0.260	146	0.066	900	0.409	2200
RAD51D PTV	1059	0.342	130	0.042	1022	0.330	20	0.006	869	0.280	3100
BARD1 PTV	1473	0.460	37	0.012	258	0.081	152	0.048	1280	0.400	3200
BRCA1 MSV	736	0.210	152	0.043	499	0.143	328	0.094	1785	0.510	3500
BRCA2 MSV	1067	0.410	264	0.102	893	0.343	46	0.018	330	0.127	2600
TP53 PTV MSV	819	0.546	380	0.253	266	0.177	19	0.013	16	0.011	1500
Total	1248191		273413		324108		136308		255580		2237600
**>60 years**											
Non-carriers	881913	0.621	155077	0.109	185440	0.131	66920	0.047	130450	0.092	1419800
BRCA1 PTV	950	0.238	121	0.030	688	0.172	267	0.067	1974	0.494	4000
BRCA2 PTV	5978	0.463	1411	0.109	2394	0.186	664	0.051	2453	0.190	12900
ATM PTV	4185	0.492	618	0.073	3021	0.355	239	0.028	437	0.051	8500
CHEK2 PTV	12431	0.644	2617	0.136	2482	0.129	694	0.036	1076	0.056	19300
PALB2 PTV	2974	0.431	902	0.131	1490	0.216	424	0.061	1110	0.161	6900
RAD51C PTV	1022	0.639	16	0.010	102	0.064	111	0.069	349	0.218	1600
RAD51D PTV	441	0.441	8	0.008	291	0.291	0	0.000	260	0.260	1000
BARD1 PTV	594	0.396	25	0.017	62	0.041	215	0.143	604	0.403	1500
BRCA1 MSV	200	0.286	2	0.003	140	0.200	39	0.056	319	0.456	700
BRCA2 MSV	56	0.140	29	0.073	185	0.463	24	0.060	106	0.265	400
TP53 PTV MSV	147	0.134	353	0.321	400	0.364	100	0.091	100	0.091	1100
Total	910891		161179		196695		69697		139238		1477700

PTV, protein truncating variants; MSV, missense variants, Total number over 100 imputations. The results represent the average proportion (over all 100 imputations) of all tumors of a particular subtype and age group. For some gene, subtype and age combinations data are limited, and therefore frequency is imprecise. MICE Imputation was carried out as described in the Methods and intrinsic subtypes constructed for each imputed data-set. These results are also shown in eFigures 2-5

© 2022 Breast Cancer Association Consortium. *JAMA Oncol.*

eTable 7: Prevalence of PTV and MSV in breast cancer susceptibility genes by intrinsic subtypes of breast cancer among women of different age groups at diagnosis

	Non-Carriers	*ATM PTV*	*BARD1 PTV*	*BRCA1 PTV*	*BRCA2 PTV*	*CHEK2 PTV*	*PALB2 PTV*	*RAD51C PTV*	*RAD51D PTV*	*BRCA1 MSV*	*BRCA2 MSV*	*TP53 PTV MSV*
**40 years**												
HR+HER2-lowgrade	0.8958	0.0055	0.0018	0.0112	0.0438	0.0293	0.0065	0.0002	0.0000	0.0018	0.0018	0.0022
HR+HER2+	0.9010	0.0034	0.0005	0.0102	0.0399	0.0224	0.0073	0.0001	0.0001	0.0009	0.0005	0.0137
HR+HER2-highgrade	0.8430	0.0131	0.0005	0.0347	0.0561	0.0257	0.0112	0.0008	0.0027	0.0048	0.0060	0.0014
HR-HER2+	0.9057	0.0008	0.0010	0.0325	0.0195	0.0189	0.0036	0.0005	0.0007	0.0002	0.0015	0.0151
TN	0.7266	0.0016	0.0039	0.1797	0.0453	0.0052	0.0073	0.0056	0.0041	0.0158	0.0019	0.0031
**41-60 years**												
HR+HER2-lowgrade	0.9523	0.0063	0.0012	0.0034	0.0129	0.0164	0.0040	0.0004	0.0008	0.0006	0.0009	0.0007
HR+HER2+	0.9504	0.0057	0.0001	0.0023	0.0121	0.0190	0.0068	0.0001	0.0005	0.0006	0.0010	0.0014
HR+HER2-highgrade	0.9018	0.0129	0.0008	0.0132	0.0315	0.0181	0.0118	0.0018	0.0032	0.0015	0.0028	0.0008
HR-HER2+	0.9518	0.0028	0.0011	0.0130	0.0073	0.0126	0.0073	0.0011	0.0001	0.0024	0.0003	0.0001
TN	0.8833	0.0034	0.0050	0.0528	0.0234	0.0071	0.0097	0.0035	0.0034	0.0070	0.0013	0.0001
**60 years**												
HR+HER2-lowgrade	0.9682	0.0046	0.0007	0.0010	0.0066	0.0136	0.0033	0.0011	0.0005	0.0002	0.0001	0.0002
HR+HER2+	0.9621	0.0038	0.0002	0.0008	0.0088	0.0162	0.0056	0.0001	0.0000	0.0000	0.0002	0.0022
HR+HER2-highgrade	0.9428	0.0154	0.0003	0.0035	0.0122	0.0126	0.0076	0.0005	0.0015	0.0007	0.0009	0.0020
HR-HER2+	0.9602	0.0034	0.0031	0.0038	0.0095	0.0100	0.0061	0.0016	0.0000	0.0006	0.0003	0.0014
TN	0.9369	0.0031	0.0043	0.0142	0.0176	0.0077	0.0080	0.0025	0.0019	0.0023	0.0008	0.0007

PTV, protein truncating variants; MSV, missense variants; HR, hormone receptor; HER2, human epidermal growth factor receptor 2; TN, triple-negative; OR, Odds Ratio; CI, Confidence
MICE Imputation was carried out as described in the Methods and intrinsic subtypes constructed for each imputed data-set. The histogram represents the average proportion (over all 100 imputations). For some gene, subtype and age combinations data are limited, and therefore frequency is imprecise.
These results are also shown in eFigures 7-9; numbers underlying the proportions are shown in eTable10

© 2022 Breast Cancer Association Consortium. *JAMA Oncol.*

eTable 8: Odds ratios for association between PTV and MSV carrier status and intrinsic subtypes refined by PR expression

Subtype	OR [a]	L95CI	U95CI	p-value	OR [a]	L95CI	U95CI	p-value
**Gene**		***ATM PTV***				***RAD51C PTV***		
**HR+HER2-lowgrade**	**1.97**	**1.52**	**2.55**	**2.61E-07**	**1.17**	**0.61**	**2.25**	**0.643**
HR+(ER+PR+)HER2-lowgrade	2.03	1.55	2.67	3.77E-07	1.09	0.53	2.25	0.813
HR+(ER+PR-)HER2-lowgrade	1.75	0.94	3.23	0.076	1.58	0.41	6.09	0.505
HR+(ER-PR+)HER2-lowgrade	1.56	0.56	4.41	0.397	0.01	NA	NA	0.798
**HR+HER2+**	**1.66**	**0.93**	**2.95**	**0.085**	**0.02**	**NA**	**NA**	**0.840**
HR+(ER+PR+)HER2+	1.78	0.92	3.45	0.086	0.01	NA	NA	0.744
HR+(ER+PR-)HER2+	1.56	0.56	4.41	0.397	0.01	NA	NA	0.798
HR+(ER-PR+)HER2+	0.01	NA	NA	0.774	0.03	NA	NA	0.856
**HR+HER2-highgrade**	**4.99**	**3.68**	**6.76**	**3.14E-25**	**2.01**	**0.82**	**4.93**	**0.129**
HR+(ER+PR+)HER2-highgrade	5.37	3.83	7.51	1.16E-22	2.14	0.81	5.66	0.124
HR+(ER+PR-)HER2-highgrade	4.30	2.26	8.16	8.33E-06	0.02	NA	NA	0.796
HR+(ER-PR+)HER2-highgrade	2.61	0.65	10.54	0.177	6.11	0.86	43.51	0.071
**PR-pos vs PR-neg (adjusted) [b]**	**1.16**	**0.74**	**1.81**	**0.521**	**1.16**	**0.28**	**4.73**	**0.838**
**Gene**		***BARD1 PTV***				***RAD51D PTV***		
**HR+HER2-lowgrade**	**2.03**	**1.12**	**3.71**	**0.020**	**1.29**	**0.65**	**2.53**	**0.466**
HR+(ER+PR+)HER2-lowgrade	1.99	1.04	3.81	0.038	1.09	0.51	2.33	0.825
HR+(ER+PR-)HER2-lowgrade	1.64	0.34	7.83	0.538	1.95	0.51	7.42	0.330
HR+(ER-PR+)HER2-lowgrade	0.02	NA	NA	0.817	1.74	0.24	12.97	0.587
**HR+HER2+**	**0.07**	**NA**	**NA**	**0.873**	**0.50**	**0.07**	**3.56**	**0.486**
HR+(ER+PR+)HER2+	0.04	NA	NA	0.770	0.01	NA	NA	0.671
HR+(ER+PR-)HER2+	0.02	NA	NA	0.817	1.74	0.24	12.97	0.587
HR+(ER-PR+)HER2+	0.06	NA	NA	0.871	0.05	NA	NA	0.837
**HR+HER2-highgrade**	**1.08**	**0.22**	**5.46**	**0.921**	**4.82**	**2.33**	**9.97**	**2.26E-05**
HR+(ER+PR+)HER2-highgrade	0.93	0.14	6.14	0.943	4.53	1.97	10.44	0.0004
HR+(ER+PR-)HER2-highgrade	0.10	NA	NA	0.865	4.22	0.09	203.45	0.467
HR+(ER-PR+)HER2-highgrade	0.39	NA	NA	0.947	6.56	0.89	48.24	0.064
**PR-pos vs PR-neg (adjusted)**	**1.23**	**0.30**	**5.11**	**0.777**	**0.64**	**0.23**	**1.76**	**0.385**
**Gene**		***BRCA1 PTV***				***BRCA1 MSV***		
**HR+HER2-lowgrade**	**3.26**	**2.21**	**4.80**	**2.07E-09**	**7.15**	**2.20**	**23.31**	**0.001**
HR+(ER+PR+)HER2-lowgrade	2.82	1.85	4.30	1.60E-06	5.14	1.38	19.15	0.015
HR+(ER+PR-)HER2-lowgrade	5.60	2.79	11.25	1.26E-06	13.87	2.72	70.80	0.002
HR+(ER-PR+)HER2-lowgrade	4.88	1.97	12.12	0.0006	0.27	NA	NA	0.910
**HR+HER2+**	**2.27**	**1.16**	**4.45**	**0.017**	**4.20**	**0.53**	**33.53**	**0.176**
HR+(ER+PR+)HER2+	1.40	0.51	3.82	0.513	4.38	0.51	37.77	0.180
HR+(ER+PR-)HER2+	4.88	1.97	12.12	0.0006	0.27	NA	NA	0.910
HR+(ER-PR+)HER2+	0.20	NA	NA	0.864	1.49	NA	NA	0.970
**HR+HER2-highgrade**	**13.50**	**9.16**	**19.90**	**1.76E-39**	**23.71**	**6.70**	**83.94**	**9.19E-07**
HR+(ER+PR+)HER2-highgrade	8.22	5.01	13.48	6.92E-17	8.51	0.54	133.64	0.128
HR+(ER+PR-)HER2-highgrade	26.60	15.45	45.79	2.59E-32	47.84	9.90	231.21	1.50E-06
HR+(ER-PR+)HER2-highgrade	32.89	15.94	67.86	3.30E-21	99.96	17.24	579.71	2.83E-07

**PR-pos vs PR-neg (adjusted)**	**0.41**	**0.27**	**0.64**	**7.05E-05**	**0.43**	**0.15**	**1.24**	**0.117**
Gene	*BRCA2 PTV*				*BRCA2 MSV*			
**HR+HER2-lowgrade**	**5.05**	**4.03**	**6.33**	**4.12E-45**	**3.73**	**1.46**	**9.56**	**0.006**
HR+(ER+PR+)HER2-lowgrade	4.74	3.74	6.01	5.13E-38	3.36	1.22	9.21	0.019
HR+(ER+PR-)HER2-lowgrade	7.27	5.00	10.56	2.45E-25	6.42	1.49	27.61	0.013
HR+(ER-PR+)HER2-lowgrade	4.80	2.43	9.49	6.48E-06	7.11	0.91	55.34	0.061
**HR+HER2+**	**5.28**	**3.73**	**7.45**	**4.26E-21**	**4.02**	**0.87**	**18.59**	**0.075**
HR+(ER+PR+)HER2+	5.23	3.54	7.71	7.17E-17	2.92	0.38	22.49	0.304
HR+(ER+PR-)HER2+	4.80	2.43	9.49	6.48E-06	7.11	0.91	55.34	0.061
HR+(ER-PR+)HER2+	7.42	2.72	20.30	9.38E-05	0.14	NA	NA	0.902
**HR+HER2-highgrade**	**11.53**	**8.92**	**14.90**	**7.88E-78**	**16.07**	**6.19**	**41.72**	**1.15E-08**
HR+(ER+PR+)HER2-highgrade	11.00	8.30	14.59	2.58E-62	15.53	5.45	44.24	2.82E-07
HR+(ER+PR-)HER2-highgrade	12.49	8.08	19.31	6.98E-30	20.53	4.36	96.78	0.0001
HR+(ER-PR+)HER2-highgrade	14.21	7.26	27.79	9.23E-15	0.27	NA	NA	0.928
**PR-pos vs PR-neg (adjusted)**	**0.77**	**0.58**	**1.01**	**0.0568**	**0.54**	**0.19**	**1.48**	**0.231**
Gene	*CHEK2 PTV*				*TP53 PTV MSV*			
**HR+HER2-lowgrade**	**2.65**	**2.25**	**3.14**	**2.27E-30**	**1.40**	**0.62**	**3.13**	**0.417**
HR+(ER+PR+)HER2-lowgrade	2.69	2.26	3.20	1.24E-28	1.28	0.53	3.11	0.583
HR+(ER+PR-)HER2-lowgrade	2.50	1.76	3.56	3.10E-07	2.11	0.41	10.94	0.375
HR+(ER-PR+)HER2-lowgrade	2.91	1.70	4.99	0.0001	7.10	1.61	31.31	0.010
**HR+HER2+**	**3.17**	**2.36**	**4.26**	**1.78E-14**	**7.14**	**3.34**	**15.28**	**4.16E-07**
HR+(ER+PR+)HER2+	3.10	2.18	4.40	2.92E-10	7.44	3.24	17.13	2.35E-06
HR+(ER+PR-)HER2+	2.91	1.70	4.99	0.0001	7.10	1.61	31.31	0.010
HR+(ER-PR+)HER2+	5.80	2.53	13.30	3.31E-05	0.09	NA	NA	0.875
**HR+HER2-highgrade**	**3.02**	**2.33**	**3.91**	**6.14E-17**	**3.40**	**1.36**	**8.46**	**0.009**
HR+(ER+PR+)HER2-highgrade	3.43	2.59	4.54	6.21E-18	2.52	0.78	8.17	0.122
HR+(ER+PR-)HER2-highgrade	1.97	1.01	3.84	0.047	7.73	2.26	26.46	0.001
HR+(ER-PR+)HER2-highgrade	1.43	0.28	7.28	0.666	0.04	NA	NA	0.844
**PR-pos vs PR-neg (adjusted)**	**1.15**	**0.87**	**1.53**	**0.331**	**0.54**	**0.24**	**1.22**	**0.141**
Gene	*PALB2 PTV*							
**HR+HER2-lowgrade**	**3.39**	**2.35**	**4.89**	**6.20E-11**				
HR+(ER+PR+)HER2-lowgrade	3.16	2.15	4.65	4.34E-09				
HR+(ER+PR-)HER2-lowgrade	4.90	2.66	9.05	3.65E-07				
HR+(ER-PR+)HER2-lowgrade	5.69	2.21	14.68	0.0003				
**HR+HER2+**	**5.70**	**3.35**	**9.70**	**1.37E-10**				
HR+(ER+PR+)HER2+	5.48	3.03	9.93	2.00E-08				
HR+(ER+PR-)HER2+	5.69	2.21	14.68	0.0003				
HR+(ER-PR+)HER2+	6.98	1.11	43.74	0.038				
**HR+HER2-highgrade**	**9.43**	**6.24**	**14.25**	**1.53E-26**				
HR+(ER+PR+)HER2-highgrade	8.72	5.50	13.83	3.20E-20				
HR+(ER+PR-)HER2-highgrade	9.21	4.34	19.56	7.68E-09				
HR+(ER-PR+)HER2-highgrade	17.78	7.22	43.81	3.96E-10				
**PR-pos vs PR-neg (adjusted)**	**0.80**	**0.52**	**1.24**	**0.323**				

PTV, protein truncating variants; MSV, missense variants; HR, Hormone receptor; ER, estrogen receptor; PR, progesterone receptor; HER2, human epidermal growth factor receptor 2; OR, Odds Ratio; CI, Confidence Intervals

[a]OR for association with intrinsic subtype (bold) and according to ER and PR status- multinomial logistic regression with controls as baseline, adjusted for country and age [b]OR for association with PR status adjusted by intrinsic subtypes, as described in the eMethods.

eTable 9: Odds ratios for association between PTV and MSV carrier status and intrinsic subtypes of breast cancer following imputation using an EM algorithm.

	**Intrinsic subtypes**			
**Gene**	OR	L95CI	U95CI	p-value
ATM PTV				
HR+HER2-lowgrade	1.88	1.45	2.44	2.05E-06
HR+HER2+	1.60	0.89	2.85	0.114
HR+HER2-highgrade	4.93	3.65	6.66	3.27E-25
HR-HER2+	1.10	0.44	2.74	0.845
TN	0.87	0.42	1.8	0.704
BRCA1 PTV				
HR+HER2-lowgrade	2.83	1.9	4.22	3.44E-07
HR+HER2+	2.88	1.44	5.77	2.89E-03
HR+HER2-highgrade	12.53	8.46	18.57	2.01E-36
HR-HER2+	8.51	4.87	14.89	5.88E-14
TN	55.4	40.6	75.61	2.69E-141
BRCA2 PTV				
HR+HER2-lowgrade	4.70	3.73	5.9	6.13E-40
HR+HER2+	6.02	4.28	8.45	3.62E-25
HR+HER2-highgrade	10.97	8.47	14.21	1.84E-73
HR-HER2+	2.15	1.09	4.23	2.70E-02
TN	9.75	7.4	12.85	8.77E-59
CHEK2 PTV				
HR+HER2-lowgrade	2.61	2.2	3.08	3.44E-29
HR+HER2+	3.01	2.22	4.07	9.91E-13
HR+HER2-highgrade	2.93	2.24	3.83	4.84E-15
HR-HER2+	2.33	1.51	3.62	0.0001
TN	0.94	0.58	1.53	0.811
PALB2 PTV				
HR+HER2-lowgrade	3.04	2.09	4.42	5.65E-09
HR+HER2+	5.99	3.57	10.05	1.22E-11
HR+HER2-highgrade	9.60	6.42	14.36	3.75E-28
HR-HER2+	4.86	2.5	9.43	3.08E-06
TN	7.28	4.67	11.37	2.38E-18
BRCA1 MSV				
HR+HER2-lowgrade	5.88	1.72	20.03	0.0046
HR+HER2+	8.31	1.09	63.09	0.04
HR+HER2-highgrade	22.13	5.99	81.8	3.42E-06
HR-HER2+	14.06	2.61	75.61	2.08E-03
TN	73.07	25.27	211.32	2.35E-15
TP53 MSV				
HR+HER2-lowgrade	1.21	0.52	2.82	0.66
HR+HER2+	7.40	3.49	15.72	1.87E-07
HR+HER2-highgrade	3.38	1.37	8.32	0.008
HR-HER2+	5.61	2.01	15.62	0.001
TN	1.55	0.38	6.35	0.544

PTV, protein truncating variants; MSV, missense variants; HR, Hormone receptor; HER2, human epidermal growth factor receptor 2; TN, triple negative; OR, Odds Ratio; CI, Confidence Intervals. Polytomous logistic regression was carried out adjusting for age as a continuous variable and country, using the EM algorithm for imputation as implemented in the TOP program (Online eMethods)

eFigure 1. Case-only analysis of phenotypic markers and prognostic features by gene (complete case analysis) Case-only logistic or multinomial logistic regression analyses adjusted for age at diagnosis/interview and country as described in the Methods. Odds Ratio and Confidence Intervals are shown. PTV, protein truncating variants; MSV, missense variants; ER, estrogen receptor; PR, progesterone receptor, HER2; human epidermal growth factor receptor 2; LN, lymph node.

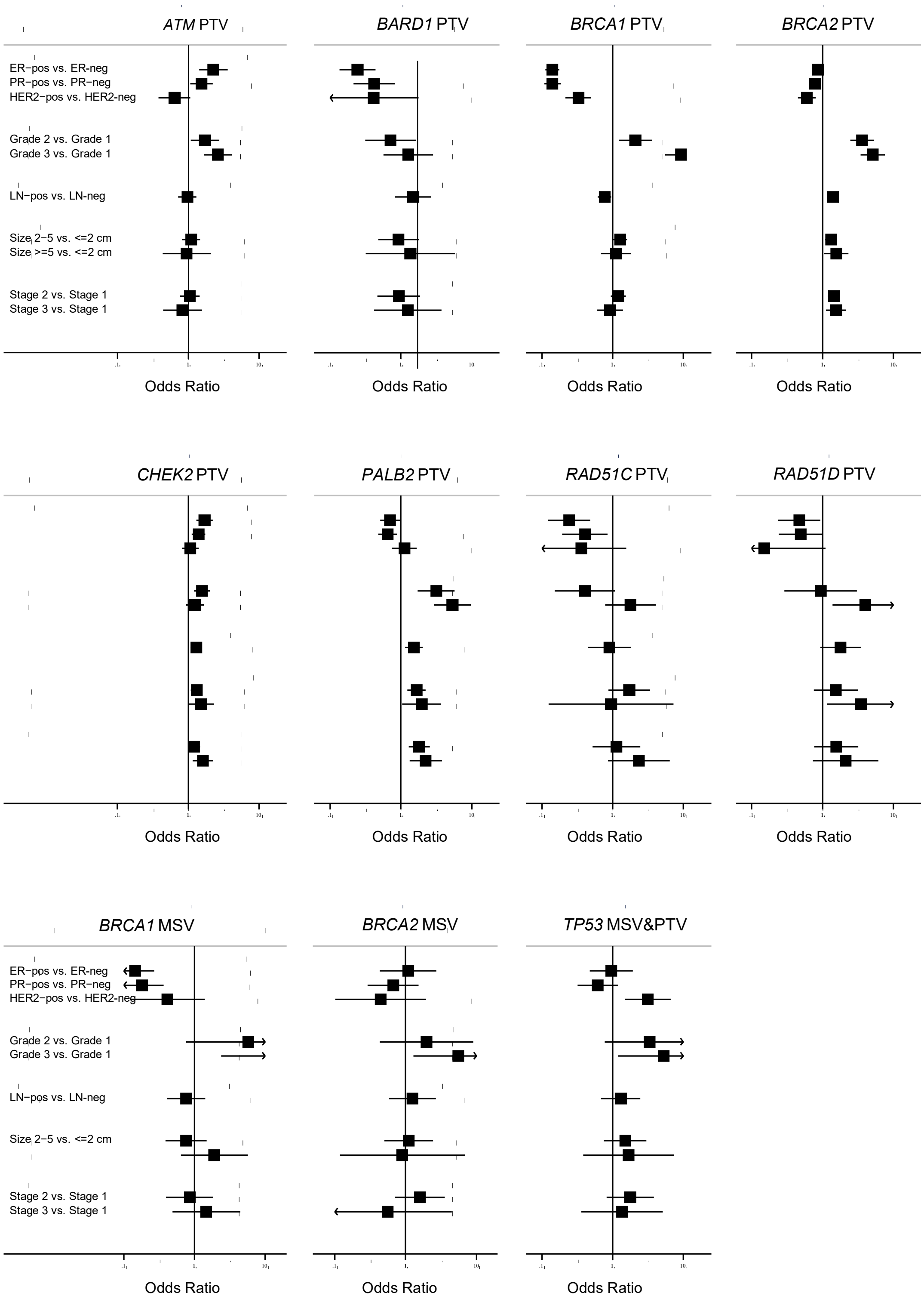
ATM PTV
BARD1 PTV
BRCA1 PTV
BRCA2 PTV
ER−pos vs. ER-neg
PR−pos vs. PR−neg
HER2−pos vs. HER2-neg
Grade 2 vs. Grade 1
Grade 3 vs. Grade 1
LN−pos vs. LN-neg
Size 2−5 vs. <=2 cm
Size >=5 vs. <=2 cm
Stage 2 vs. Stage 1
Stage 3 vs. Stage 1
Odds Ratio
CHEK2 PTV
PALB2 PTV
RAD51C PTV
RAD51D PTV
BRCA1 MSV
BRCA2 MSV
TP53 MSV&PTV

eFigure 2. Frequency distribution of intrinsic subtypes among noncarriers and carriers of PTVs and MSVs in the 9 genes. MICE Imputation was carried out as described in the Methods and intrinsic subtypes constructed for each imputed data-set. The histogram represents the average proportion (over all 100 imputations) of all tumors of a particular subtype. For some genes and subtypes, data are limited, and therefore frequency is imprecise. These results are also shown in eTable 10.

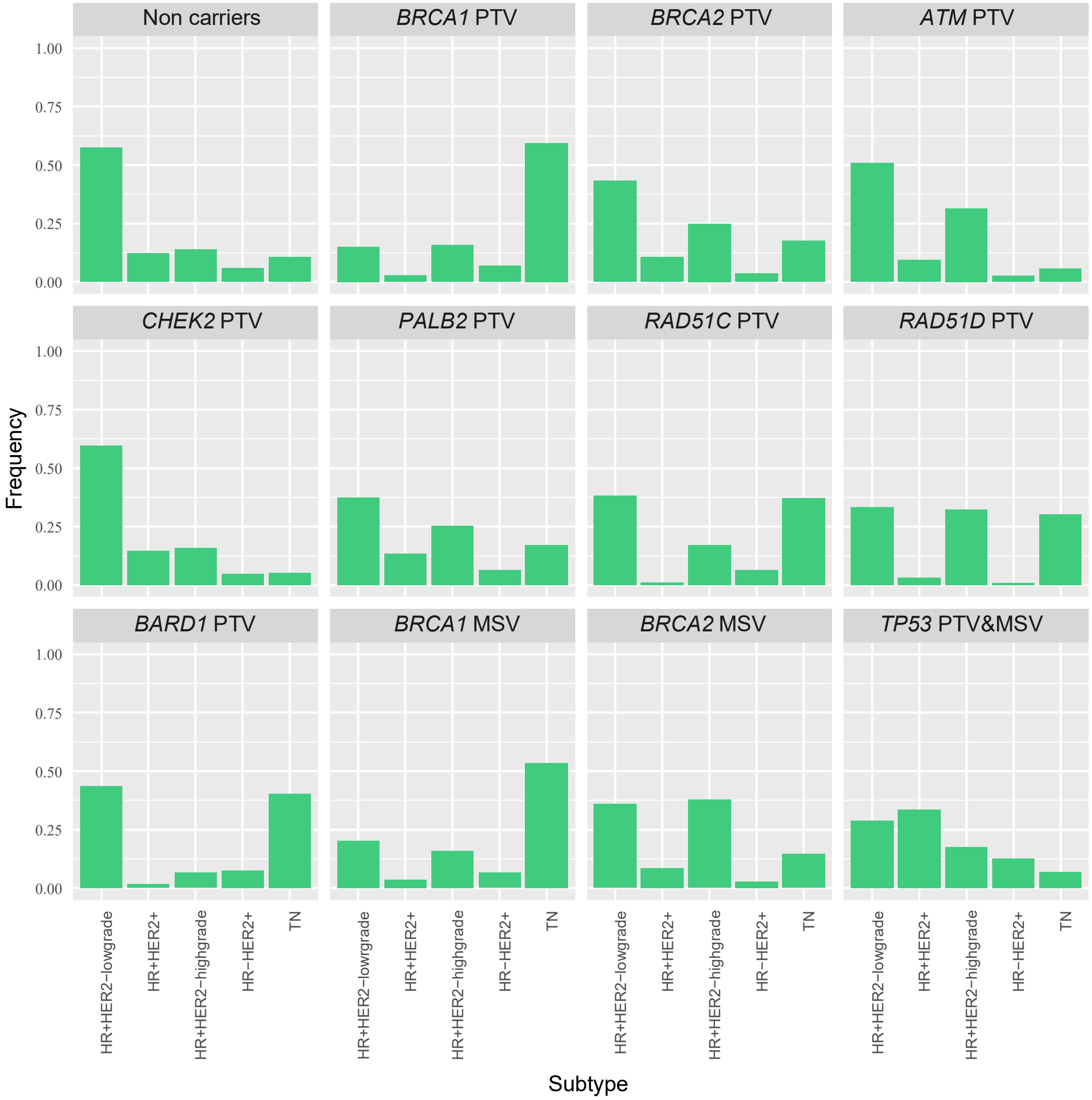

eFigure 3. Frequency distribution of intrinsic subtypes among noncarriers and carriers of PTVs and MSVs in the 9 genes, in women aged ≤40 years.

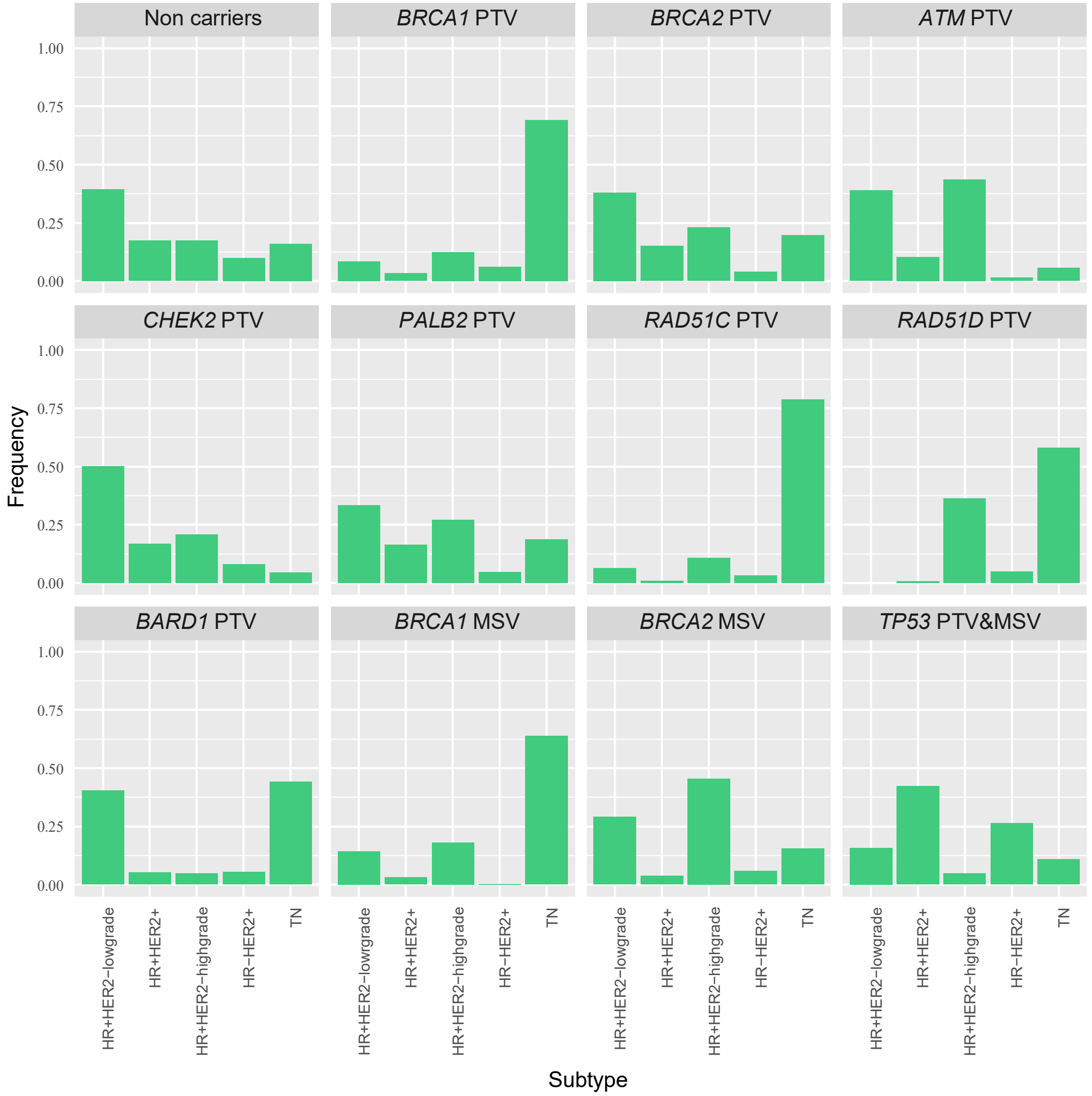

eFigure 4. Frequency distribution of intrinsic subtypes among noncarriers and carriers of PTVs and MSVs in the 9 genes, in women aged 41-60 years.

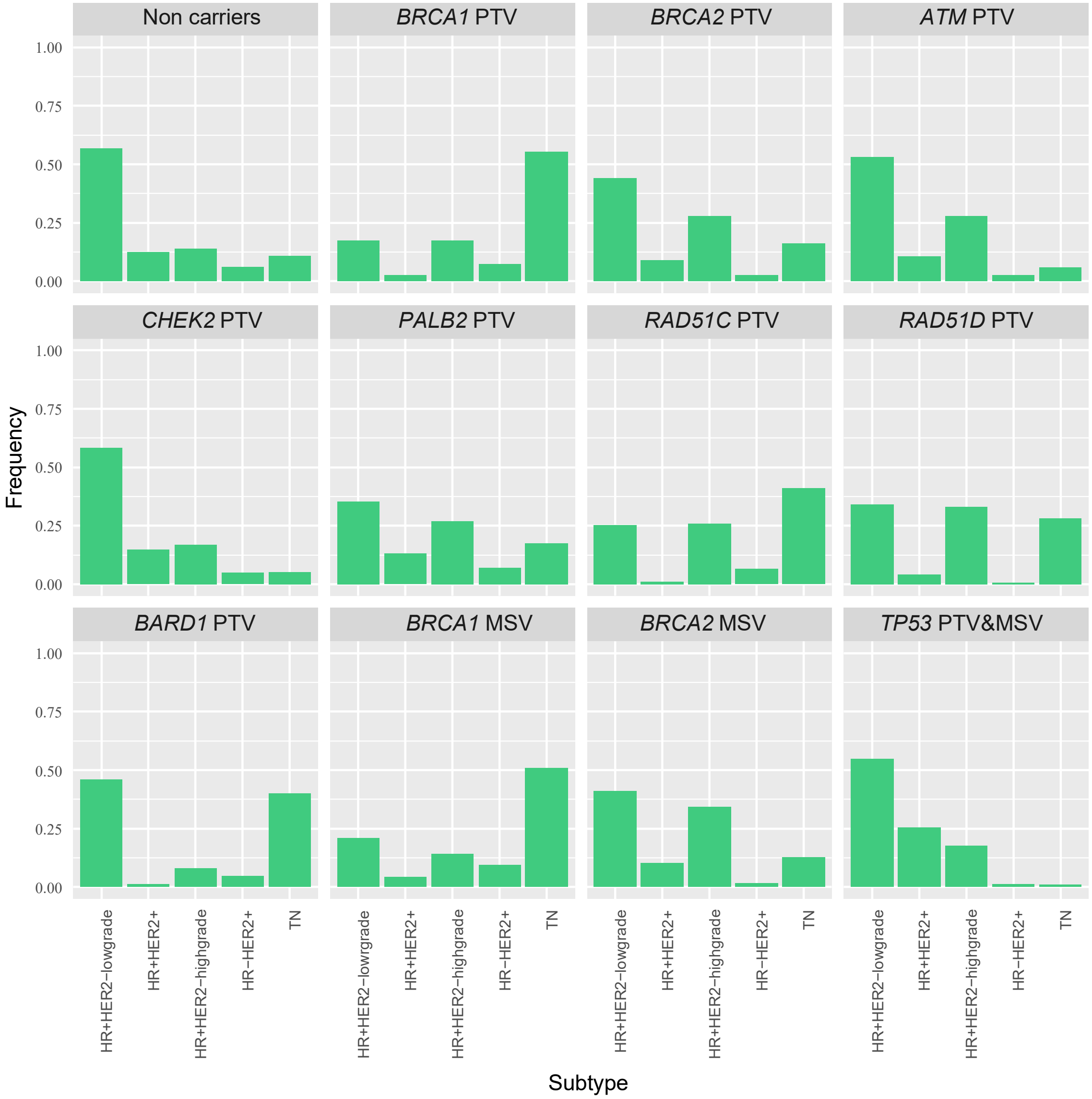

eFigure 5. Frequency distribution of intrinsic subtypes among noncarriers and carriers of PTVs and MSVs in the 9 genes, in women aged >60 years.

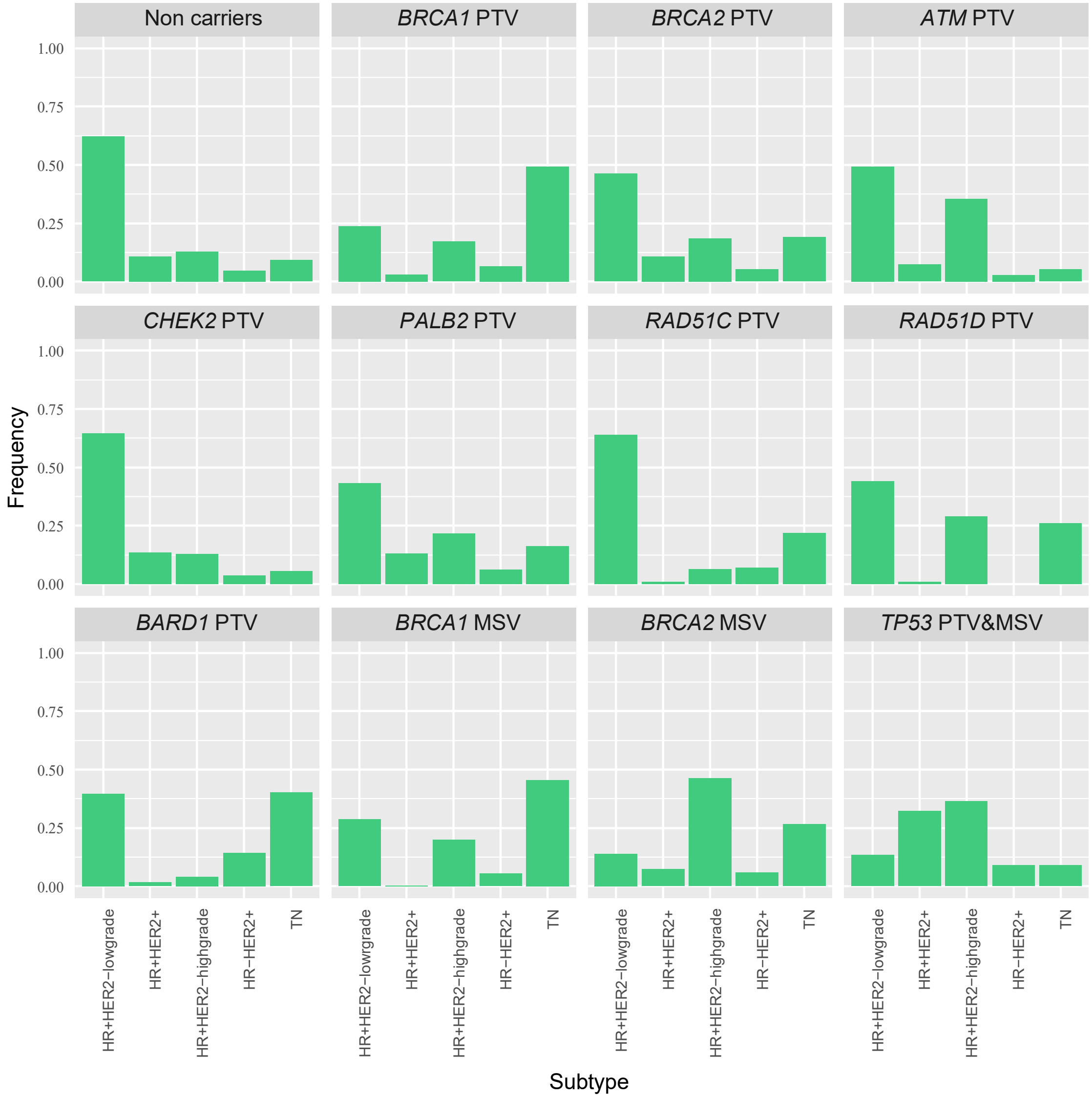

eFigure 6. Association odds ratios for MSV carrier status in *BRCA1*, *BRCA2*, *TP53* and intrinsic subtypes of breast cancer. MICE Imputation was carried out as described in the Methods and intrinsic subtypes constructed for each imputed data-set. Multinomial logistic regression as carried out with intrinsic subtypes as the outcome variable, adjusting by age at diagnosis/interview and country and the results of these analyses were pooled. These results are also shown in eTable 9.

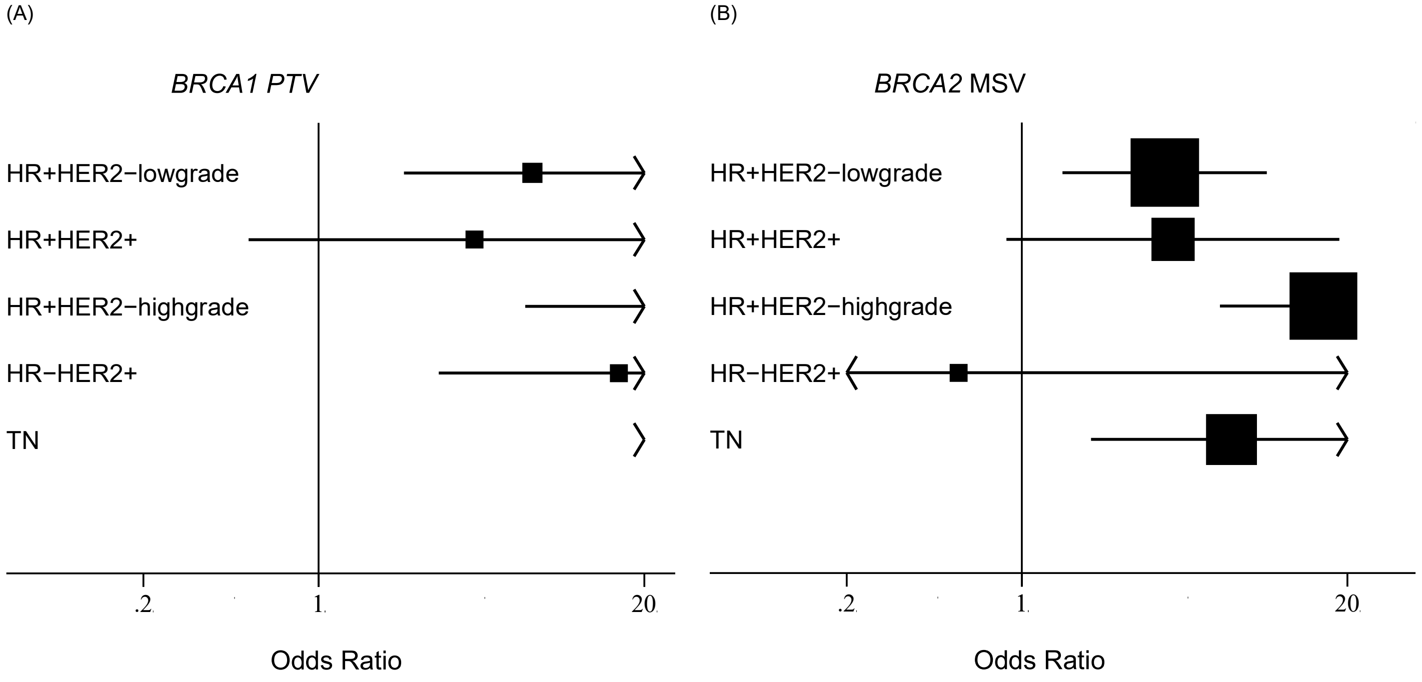

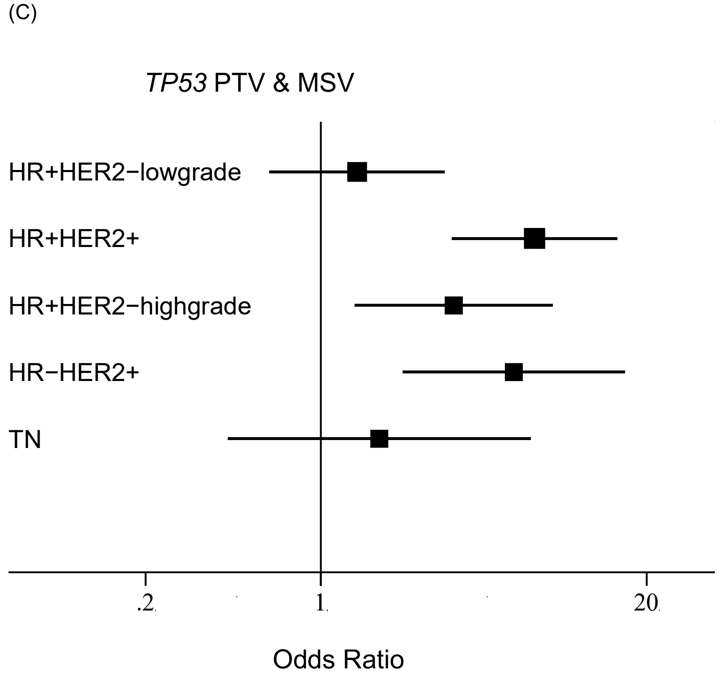

eFigure 7. Prevalence of PTV and MSV in breast cancer susceptibility genes by intrinsic subtypes of breast cancer among women aged ≤40 at diagnosis (A) HR+ HER2- low-grade (B) HR+ HER2- (C) HR+ HER2- high-grade (D) HR- HER2+ (E) TN.

(A)

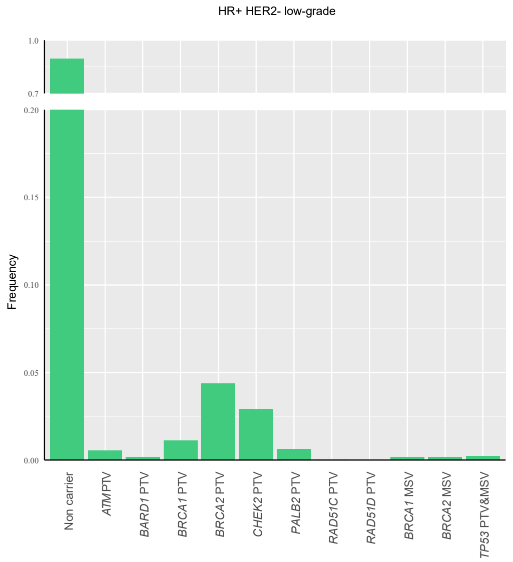

(B)

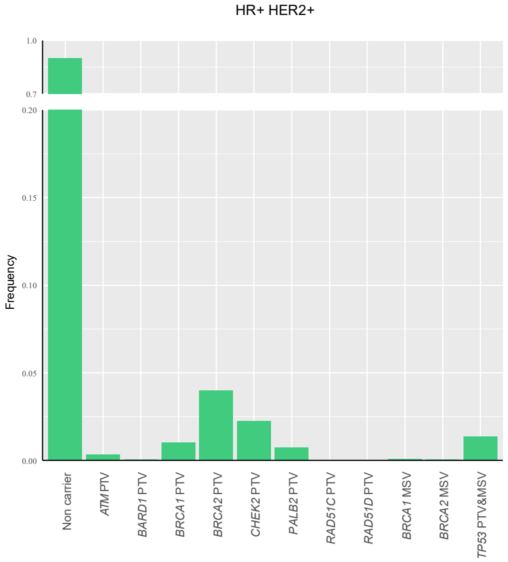

(C)

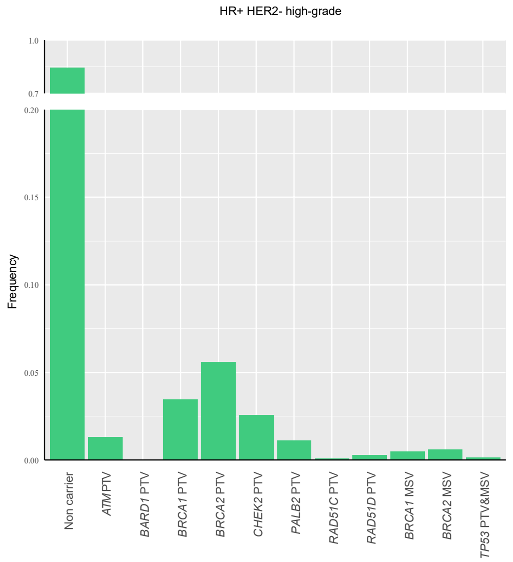

(D)

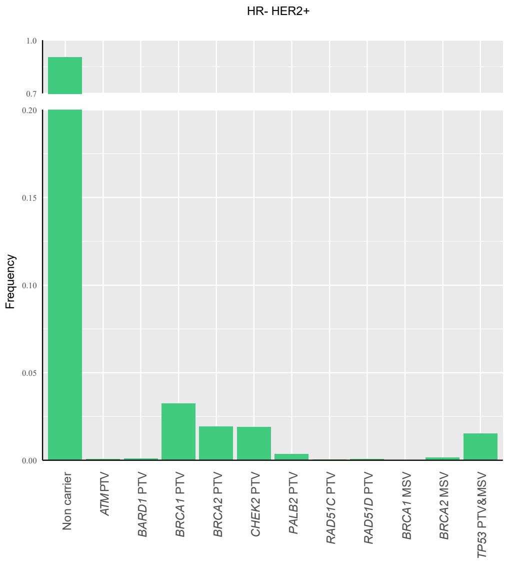

(E)

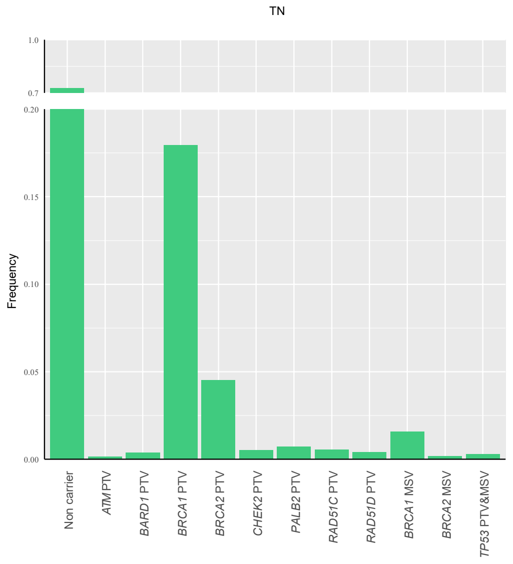

eFigure 8. Prevalence of PTV and MSV in breast cancer susceptibility genes by intrinsic subtypes of breast cancer among women aged 41-60 at diagnosis (A) HR+ HER2- low-grade (B) HR+ HER2- (C) HR+ HER2- high-grade (D) HR- HER2+ (E) TN.

(A)

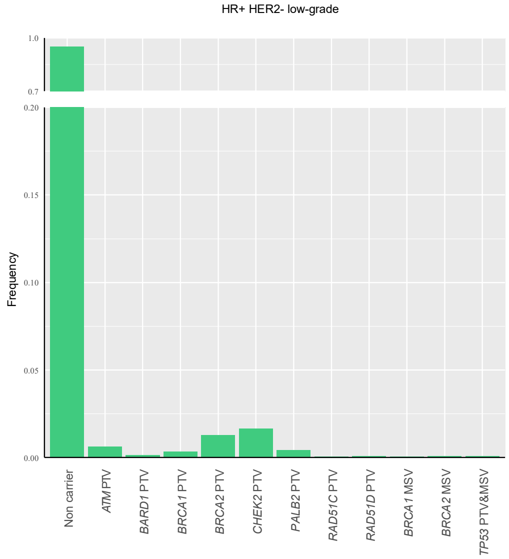

(B)

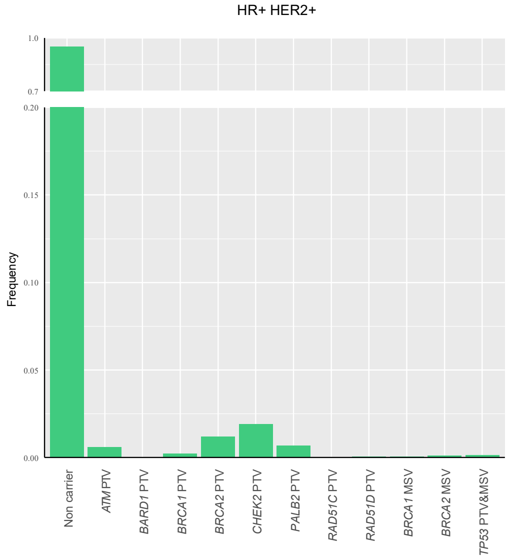

(C)

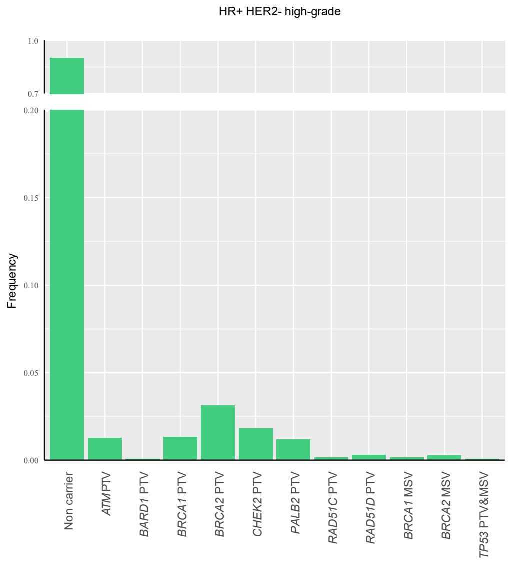

(D)

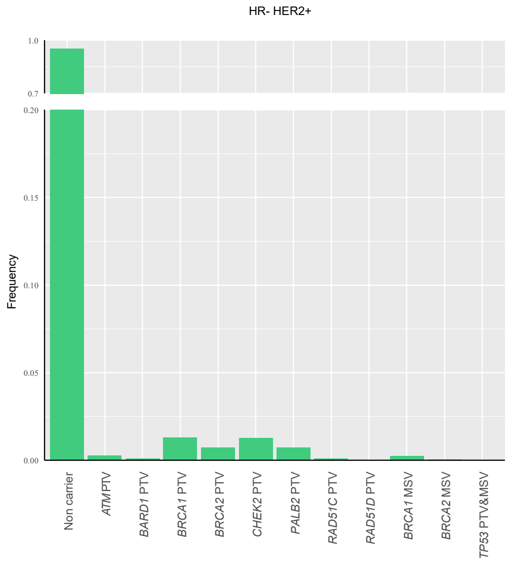

(E)

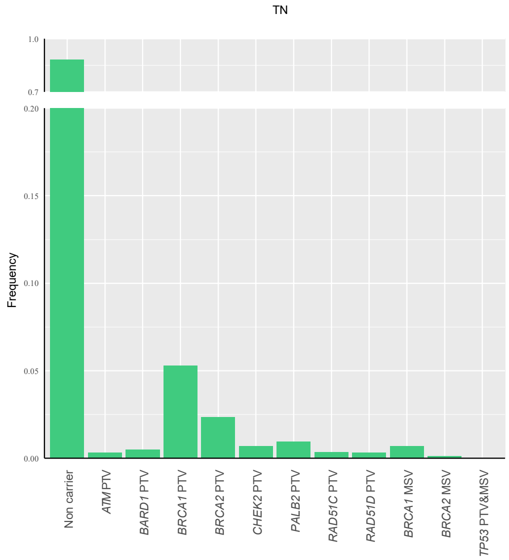

eFigure 9. Prevalence of PTV and MSV in breast cancer susceptibility genes by intrinsic subtypes of breast cancer among women aged >60 at diagnosis (A) HR+ HER2- low-grade (B) HR+ HER2- (C) HR+ HER2- high-grade (D) HR- HER2+ (E) TN.

(A)

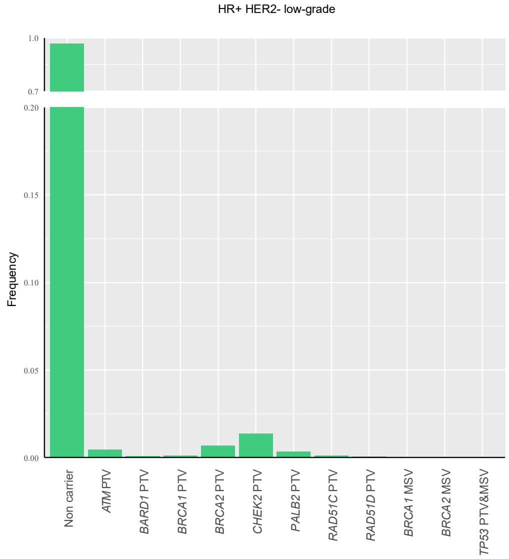

(B)

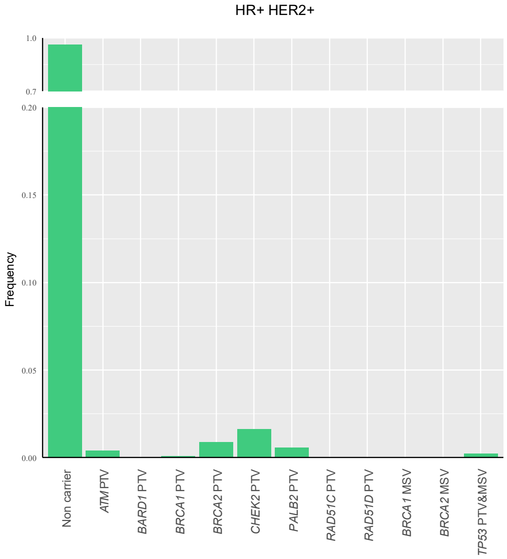

(C)

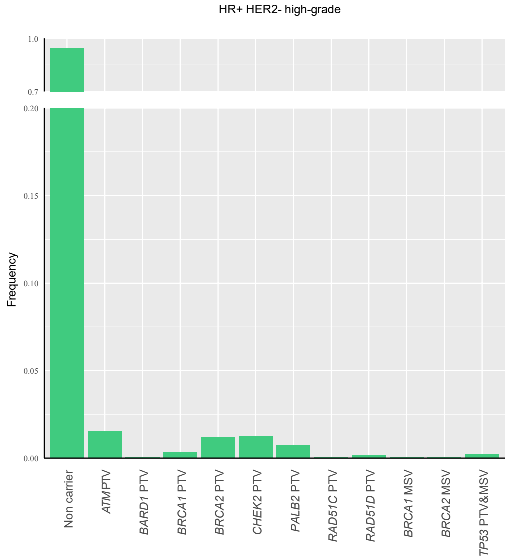

(D)

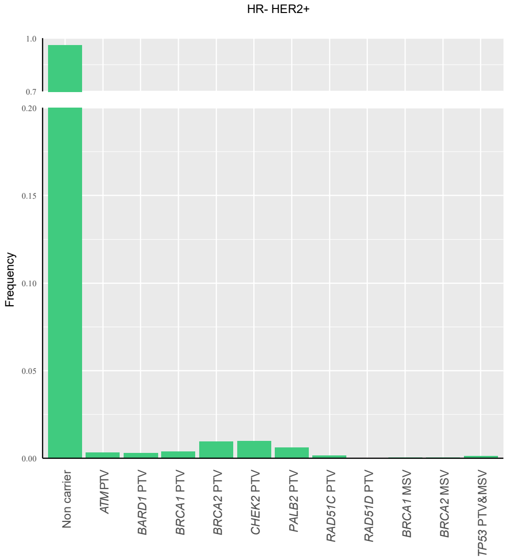

(E)

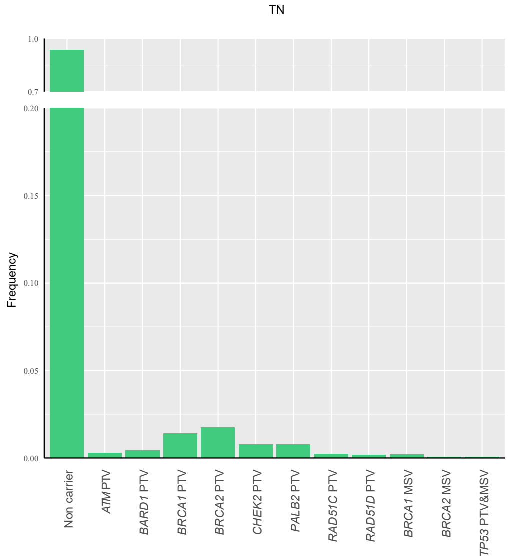

eFigure 10. Prevalence of PTV and MSV in breast cancer susceptibility genes by tumor grade among women aged ≤40 at diagnosis (A) Grade 1 (B) Grade 2 (C) Grade 3.

(A)

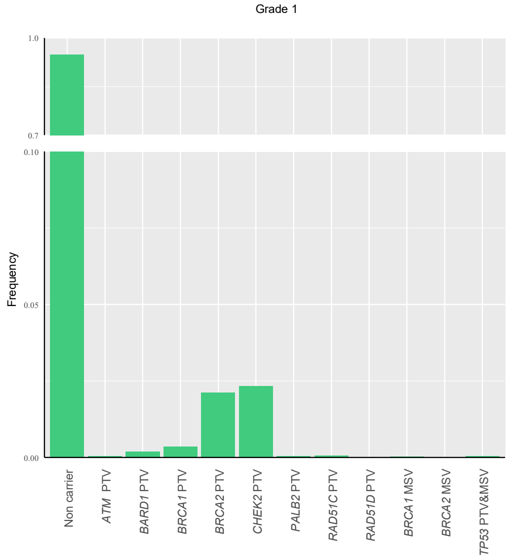

(B)

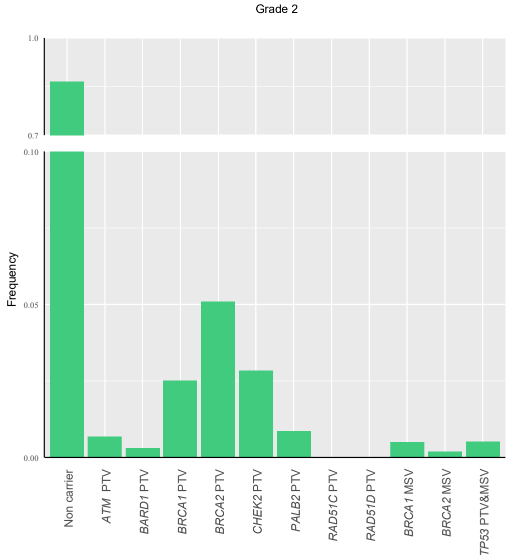

(C)

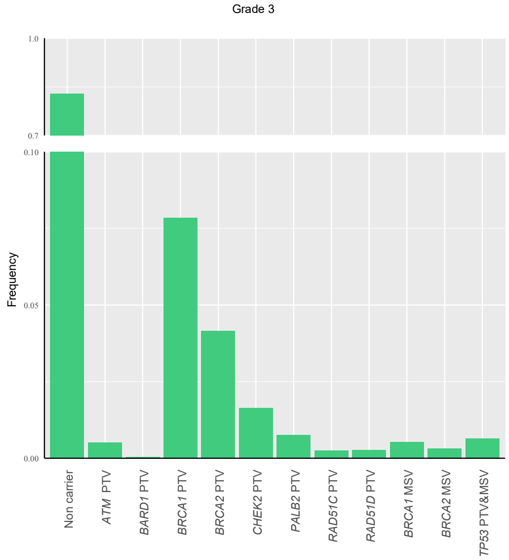

eFigure 11. Prevalence of PTV and MSV in breast cancer susceptibility genes by tumor grade among women aged 41-60 at diagnosis (A) Grade 1 (B) Grade 2 (C) Grade 3.

(A)

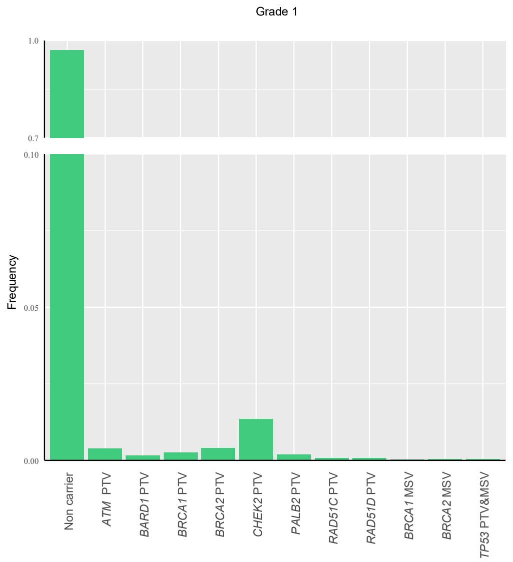

(B)

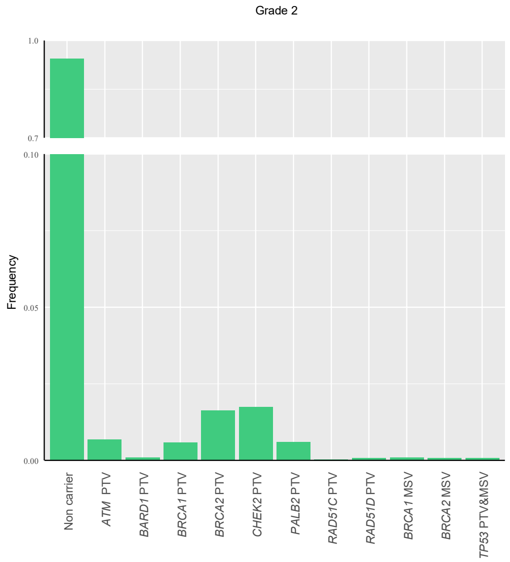

(C)

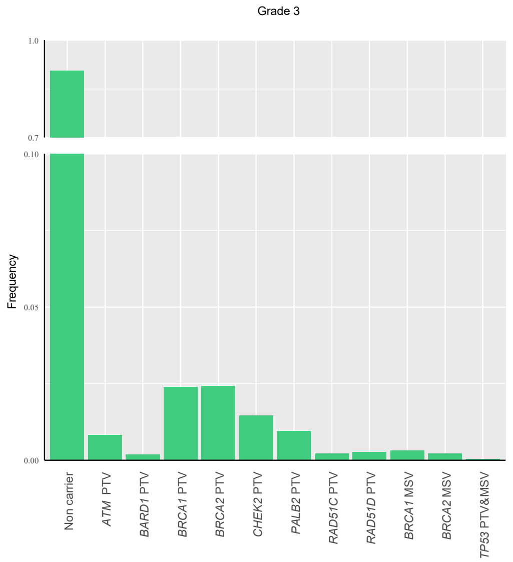

eFigure 12. Prevalence of PTV and MSV in breast cancer susceptibility genes by tumor grade among women aged >60 at diagnosis (A) Grade 1 (B) Grade 2 (C) Grade 3.

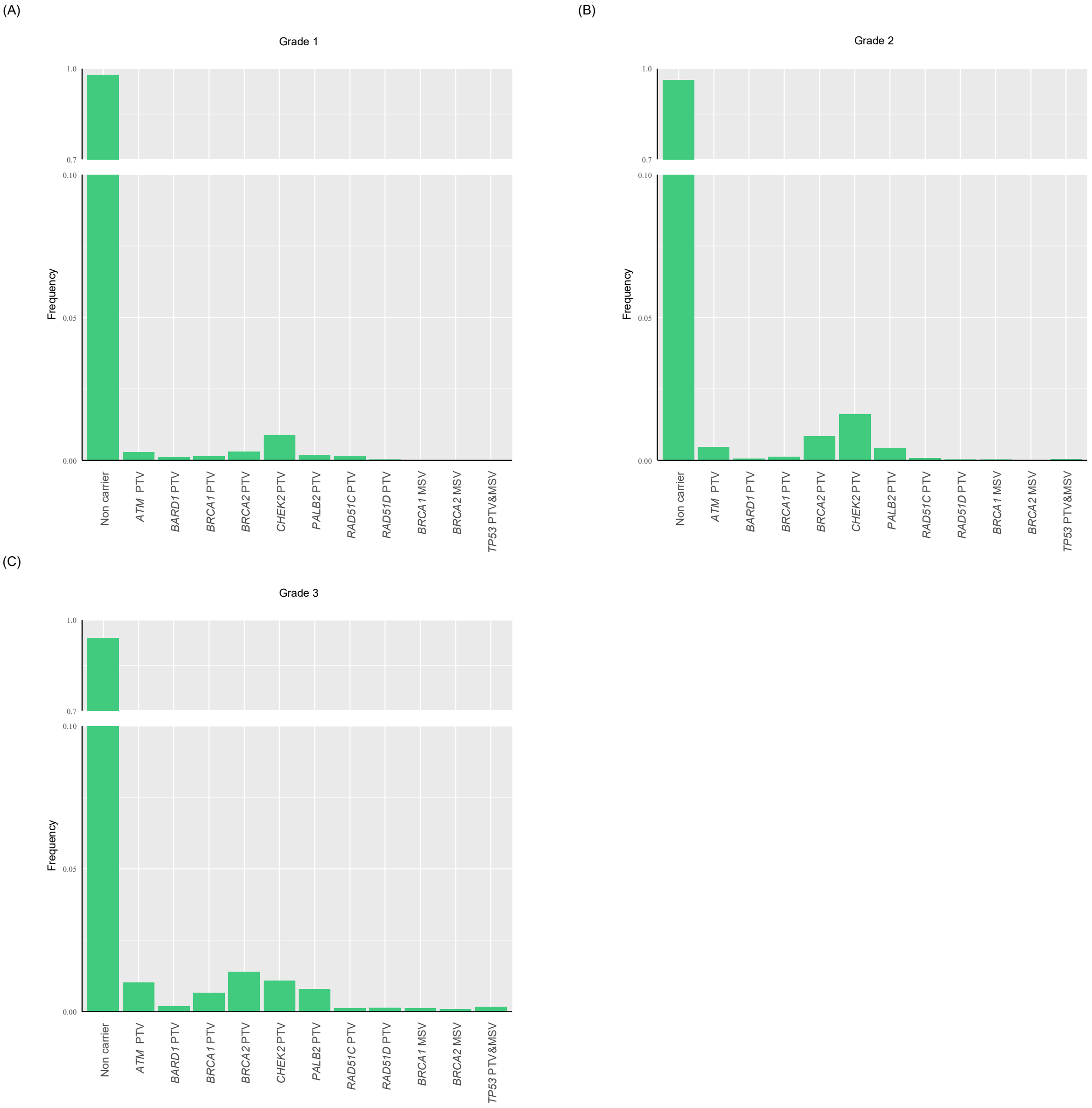

eFigure 13. Smoothed proportions of subtypes used in BOADICEA for PTVs in (A) non-carriers (B) *ATM* (C) *BARD1* (D) *BRCA1* (E) *BRCA2* (F) *CHEK2* (G) *PALB2* (H) *RAD51C* and (I) *RAD51D*. Age-specific smoothed proportions of subtypes used in BOADICEA/CanRisk were derived as described in the eMethods. These results are the same as those shown in eTable 14.

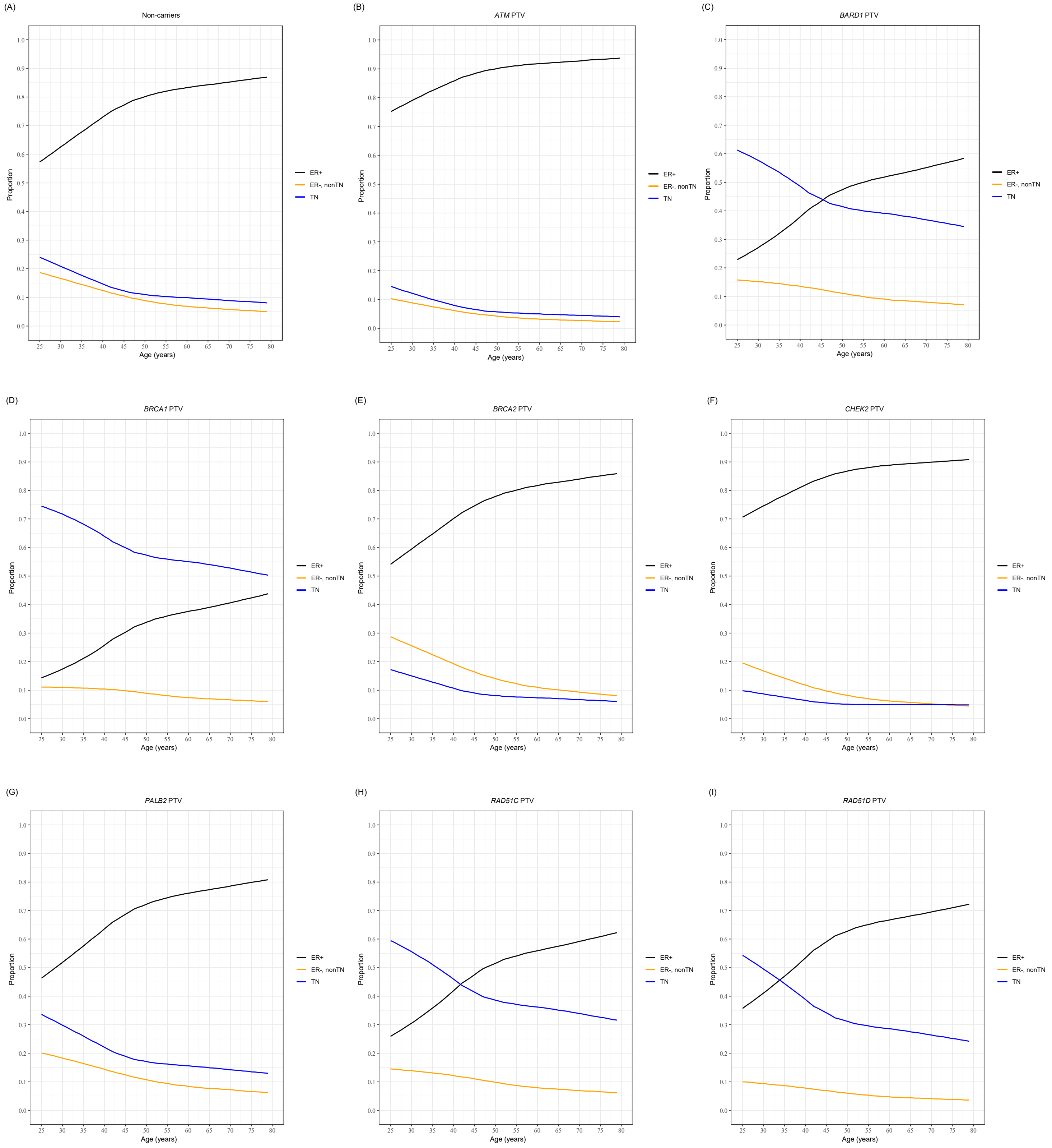

eReferences

1. Dorling L, Carvalho S, Allen J, et al. Breast Cancer Risk Genes - Association Analysis in More than 113,000 Women. *N Engl J Med.* 2021;384(5):428-439.
2. Schmidt MK, Hogervorst F, van Hien R, et al. Age- and Tumor Subtype-Specific Breast Cancer Risk Estimates for CHEK2*1100delC Carriers. *J Clin Oncol.* 2016;34(23):2750-2760
3. Broeks A, Schmidt MK, Sherman ME, et al. Low penetrance breast cancer susceptibility loci are associated with specific breast tumor subtypes: findings from the Breast Cancer Association Consortium. *Hum Mol Genet.* 2011;20(16):3289-3303.
4. Li H. Aligning sequence reads, clone sequences and assembly contigs with BWA-MEM. *arXiv*. 2013;1303:3997.
5. Lai Z, Markovets A, Ahdesmaki M, et al. VarDict: a novel and versatile variant caller for next-generation sequencing in cancer research. *Nucleic Acids Res.* 2016;44(11):e108.
6. Richards S, Aziz N, Bale S, et al. Standards and guidelines for the interpretation of sequence variants: a joint consensus recommendation of the American College of Medical Genetics and Genomics and the Association for Molecular Pathology. *Genet Med.* 2015;17(5):405-424.
7. Fortuno C, Cipponi A, Ballinger ML, et al. A quantitative model to predict pathogenicity of missense variants in the TP53 gene. *Hum Mutat.* 2019;40(6):788-800.
8. Zhang H, Zhao N, Ahearn TU, Wheeler W, García-Closas M, Chatterjee N. A mixed-model approach for powerful testing of genetic associations with cancer risk incorporating tumor characteristics. *Biostatistics.* 2020. doi: 10.1093/biostatistics/kxz065
9. Mavaddat N, Pharoah PD, Michailidou K, et al. Prediction of breast cancer risk based on profiling with common genetic variants. *J Natl Cancer Inst.* 2015;107(5): 10.1093/jnci/djv036
10. Lee AJ, Cunningham AP, Kuchenbaecker KB, Mavaddat N, Easton DF, Antoniou AC. BOADICEA breast cancer risk prediction model: updates to cancer incidences, tumour pathology and web interface. *Br J Cancer.* 2014;110(2):535-545.
11. Schmidt MK, Tollenaar RA, de Kemp SR, et al. Breast cancer survival and tumor characteristics in premenopausal women carrying the CHEK2*1100delC germline mutation. *J Clin Oncol.* 2007;25(1):64-69.
12. Michailidou K, Hall P, Gonzalez-Neira A, et al. Large-scale genotyping identifies 41 new loci associated with breast cancer risk. *Nat Genet.* 2013;45(4):353-361, 361e351-352.
13. Fasching PA, Loehberg CR, Strissel PL, et al. Single nucleotide polymorphisms of the aromatase gene (CYP19A1), HER2/neu status, and prognosis in breast cancer patients. *Breast Cancer Res Treat.* 2008;112(1):89-98.
14. Schrauder M, Frank S, Strissel PL, et al. Single nucleotide polymorphism D1853N of the ATM gene may alter the risk for breast cancer. *J Cancer Res Clin Oncol.* 2008;134(8):873-882.

15. Colleran G, McInerney N, Rowan A, et al. The TGFBR1*6A/9A polymorphism is not associated with differential risk of breast cancer. *Breast Cancer Res Treat.* 2010;119(2):437-442.
16. McInerney N, Colleran G, Rowan A, et al. Low penetrance breast cancer predisposition SNPs are site specific. *Breast Cancer Res Treat.* 2009;117(1):151-159.
17. Jiang X, Castelao JE, Chavez-Uribe E, et al. Family history and breast cancer hormone receptor status in a Spanish cohort. *PLoS One.* 2012;7(1):e29459.
18. Redondo CM, Gago-Dominguez M, Ponte SM, et al. Breast feeding, parity and breast cancer subtypes in a Spanish cohort. *PLoS One.* 2012;7(7):e40543.
19. Ali AM, Schmidt MK, Bolla MK, et al. Alcohol consumption and survival after a breast cancer diagnosis: a literature-based meta-analysis and collaborative analysis of data for 29,239 cases. *Cancer Epidemiol Biomarkers Prev.* 2014;23(6):934-945.
20. Cruz GI, Martinez ME, Natarajan L, et al. Hypothesized role of pregnancy hormones on HER2+ breast tumor development. *Breast Cancer Res Treat.* 2013;137(1):237-246.
21. Gago-Dominguez M, Castelao JE, Gude F, et al. Alcohol and breast cancer tumor subtypes in a Spanish Cohort. *Springerplus.* 2016;5:39.
22. Yang R, Dick M, Marme F, et al. Genetic variants within miR-126 and miR-335 are not associated with breast cancer risk. *Breast Cancer Res Treat.* 2011;127(2):549-554.
23. Menegaux F, Truong T, Anger A, et al. Night work and breast cancer: a population-based case-control study in France (the CECILE study). *Int J Cancer.* 2013;132(4):924-931.
24. Weischer M, Bojesen SE, Tybjaerg-Hansen A, Axelsson CK, Nordestgaard BG. Increased risk of breast cancer associated with CHEK2*1100delC. *J Clin Oncol.* 2007;25(1):57-63.
25. Milne RL, Ribas G, Gonzalez-Neira A, et al. ERCC4 associated with breast cancer risk: a two-stage case-control study using high-throughput genotyping. *Cancer Res.* 2006;66(19):9420-9427.
26. Kast K, Rhiem K, Wappenschmidt B, et al. Prevalence of BRCA1/2 germline mutations in 21 401 families with breast and ovarian cancer. *J Med Genet.* 2016;53(7):465-471.
27. Rhiem K, Engel C, Graeser M, et al. The risk of contralateral breast cancer in patients from BRCA1/2 negative high risk families as compared to patients from BRCA1 or BRCA2 positive families: a retrospective cohort study. *Breast Cancer Res.* 2012;14(6):R156.
28. Graeser MK, Engel C, Rhiem K, et al. Contralateral breast cancer risk in BRCA1 and BRCA2 mutation carriers. *J Clin Oncol.* 2009;27(35):5887-5892.
29. Engel C, Rhiem K, Hahnen E, et al. Prevalence of pathogenic BRCA1/2 germline mutations among 802 women with unilateral triple-negative breast cancer without family cancer history. *BMC Cancer.* 2018;18(1):265.
30. Pesch B, Ko Y, Brauch H, et al. Factors modifying the association between hormone-replacement therapy and breast cancer risk. *Eur J Epidemiol.* 2005;20(8):699-711.
31. Justenhoven C, Pierl CB, Haas S, et al. The CYP1B1_1358_GG genotype is associated with estrogen receptor-negative breast cancer. *Breast Cancer Res Treat.* 2008;111(1):171-177.

32. Smith BH, Campbell A, Linksted P, et al. Cohort Profile: Generation Scotland: Scottish Family Health Study (GS:SFHS). The study, its participants and their potential for genetic research on health and illness. *Int J Epidemiol.* 2013;42(3):689-700.
33. Chang-Claude J, Eby N, Kiechle M, Bastert G, Becher H. Breastfeeding and breast cancer risk by age 50 among women in Germany. *Cancer Causes Control.* 2000;11(8):687-695.
34. Dork T, Bendix R, Bremer M, et al. Spectrum of ATM gene mutations in a hospital-based series of unselected breast cancer patients. *Cancer Res.* 2001;61(20):7608-7615.
35. Syrjakoski K, Vahteristo P, Eerola H, et al. Population-based study of BRCA1 and BRCA2 mutations in 1035 unselected Finnish breast cancer patients. *J Natl Cancer Inst.* 2000;92(18):1529-1531.
36. Kilpivaara O, Bartkova J, Eerola H, et al. Correlation of CHEK2 protein expression and c.1100delC mutation status with tumor characteristics among unselected breast cancer patients. *Int J Cancer.* 2005;113(4):575-580.
37. Fagerholm R, Hofstetter B, Tommiska J, et al. NAD(P)H:quinone oxidoreductase 1 NQO1*2 genotype (P187S) is a strong prognostic and predictive factor in breast cancer. *Nat Genet.* 2008;40(7):844-853.
38. Bogdanova N, Cybulski C, Bermisheva M, et al. A nonsense mutation (E1978X) in the ATM gene is associated with breast cancer. *Breast Cancer Res Treat.* 2009;118(1):207-211.
39. Wendt C, Lindblom A, Arver B, von Wachenfeldt A, Margolin S. Tumour spectrum in non-BRCA hereditary breast cancer families in Sweden. *Hered Cancer Clin Pract.* 2015;13(1):15.
40. Margolin S, Werelius B, Fornander T, Lindblom A. BRCA1 mutations in a population-based study of breast cancer in Stockholm County. *Genet Test.* 2004;8(2):127-132.
41. Hartikainen JM, Tuhkanen H, Kataja V, et al. An autosome-wide scan for linkage disequilibrium-based association in sporadic breast cancer cases in eastern Finland: three candidate regions found. *Cancer Epidemiol Biomarkers Prev.* 2005;14(1):75-80.
42. Hartikainen JM, Tuhkanen H, Kataja V, et al. Refinement of the 22q12-q13 breast cancer--associated region: evidence of TMPRSS6 as a candidate gene in an eastern Finnish population. *Clin Cancer Res.* 2006;12(5):1454-1462.
43. Mann GJ, Thorne H, Balleine RL, et al. Analysis of cancer risk and BRCA1 and BRCA2 mutation prevalence in the kConFab familial breast cancer resource. *Breast Cancer Res.* 2006;8(1):R12.
44. Beesley J, Jordan SJ, Spurdle AB, et al. Association between single-nucleotide polymorphisms in hormone metabolism and DNA repair genes and epithelial ovarian cancer: results from two Australian studies and an additional validation set. *Cancer Epidemiol Biomarkers Prev.* 2007;16(12):2557-2565.
45. Flesch-Janys D, Slanger T, Mutschelknauss E, et al. Risk of different histological types of postmenopausal breast cancer by type and regimen of menopausal hormone therapy. *Int J Cancer.* 2008;123(4):933-941.
46. Hadjisavvas A, Loizidou MA, Middleton N, et al. An investigation of breast cancer risk factors in Cyprus: a case control study. *BMC Cancer.* 2010;10:447.
47. Giles GG, English DR. The Melbourne Collaborative Cohort Study. *IARC Sci Publ.* 2002;156:69-70.

48. Phuah SY, Looi LM, Hassan N, et al. Triple-negative breast cancer and PTEN (phosphatase and tensin homologue) loss are predictors of BRCA1 germline mutations in women with early-onset and familial breast cancer, but not in women with isolated late-onset breast cancer. *Breast Cancer Res.* 2012;14(6):R142.
49. Mariapun S, Ho WK, Kang PC, et al. Variants in 6q25.1 Are Associated with Mammographic Density in Malaysian Chinese Women. *Cancer Epidemiol Biomarkers Prev.* 2016;25(2):327-333.
50. Aure MR, Jernstrom S, Krohn M, et al. Integrated analysis reveals microRNA networks coordinately expressed with key proteins in breast cancer. *Genome Med.* 2015;7(1):21.
51. Fleischer T, Edvardsen H, Solvang HK, et al. Integrated analysis of high-resolution DNA methylation profiles, gene expression, germline genotypes and clinical end points in breast cancer patients. *Int J Cancer.* 2014;134(11):2615-2625.
52. Fleischer T, Frigessi A, Johnson KC, et al. Genome-wide DNA methylation profiles in progression to in situ and invasive carcinoma of the breast with impact on gene transcription and prognosis. *Genome Biol.* 2014;15(8):435.
53. Quigley DA, Fiorito E, Nord S, et al. The 5p12 breast cancer susceptibility locus affects MRPS30 expression in estrogen-receptor positive tumors. *Mol Oncol.* 2014;8(2):273-284.
54. John EM, Hopper JL, Beck JC, et al. The Breast Cancer Family Registry: an infrastructure for cooperative multinational, interdisciplinary and translational studies of the genetic epidemiology of breast cancer. *Breast Cancer Res.* 2004;6(4):R375-389.
55. Garcia-Closas M, Egan KM, Newcomb PA, et al. Polymorphisms in DNA double-strand break repair genes and risk of breast cancer: two population-based studies in USA and Poland, and meta-analyses. *Hum Genet.* 2006;119(4):376-388.
56. Pfeiffer RM, Park Y, Kreimer AR, et al. Risk prediction for breast, endometrial, and ovarian cancer in white women aged 50 y or older: derivation and validation from population-based cohort studies. *PLoS Med.* 2013;10(7):e1001492.
57. Evans DG, Astley S, Stavrinos P, et al. *Improvement in risk prediction, early detection and prevention of breast cancer in the NHS Breast Screening Programme and family history clinics: a dual cohort study*. Southampton (UK)2016.
58. Wedren S, Lovmar L, Humphreys K, et al. Oestrogen receptor alpha gene haplotype and postmenopausal breast cancer risk: a case control study. *Breast Cancer Res.* 2004;6(4):R437-449.
59. Lesueur F, Pharoah PD, Laing S, et al. Allelic association of the human homologue of the mouse modifier Ptprj with breast cancer. *Hum Mol Genet.* 2005;14(16):2349-2356.
60. Stevens KN, Fredericksen Z, Vachon CM, et al. 19p13.1 is a triple-negative-specific breast cancer susceptibility locus. *Cancer Res.* 2012;72(7):1795-1803.
61. Jakubowska A, Cybulski C, Szymanska A, et al. BARD1 and breast cancer in Poland. *Breast Cancer Res Treat.* 2008;107(1):119-122.
62. Jakubowska A, Jaworska K, Cybulski C, et al. Do BRCA1 modifiers also affect the risk of breast cancer in non-carriers? *Eur J Cancer.* 2009;45(5):837-842.
63. Cybulski C, Kluzniak W, Huzarski T, et al. Clinical outcomes in women with breast cancer and a PALB2 mutation: a prospective cohort analysis. *Lancet Oncol.* 2015;16(6):638-644.

64. Cybulski C, Carrot-Zhang J, Kluzniak W, et al. Germline RECQL mutations are associated with breast cancer susceptibility. *Nat Genet.* 2015;47(6):643-646.
65. Madsen MJ, Knight S, Sweeney C, et al. Reparameterization of PAM50 Expression Identifies Novel Breast Tumor Dimensions and Leads to Discovery of a Genome-Wide Significant Breast Cancer Locus at 12q15. *Cancer Epidemiol Biomarkers Prev.* 2018;27(6):644-652.
66. Camp NJ, Parry M, Knight S, et al. Fine-mapping CASP8 risk variants in breast cancer. *Cancer Epidemiol Biomarkers Prev.* 2012;21(1):176-181.
